# Beyond Platinum: Defects Abundant CoP_3_/Ni_2_P Heterostructure for Hydrogen Evolution Electrocatalysis

**DOI:** 10.1002/smsc.202000027

**Published:** 2021-03-06

**Authors:** Lijie Zhang, Linzhou Zhuang, Hongli Liu, Longzhou Zhang, Rongsheng Cai, Ning Chen, Xianfeng Yang, Zhonghua Zhu, Dongjiang Yang, Xiangdong Yao

**Affiliations:** ^1^ School of Environmental Science and Engineering State Key Laboratory of Bio-fibers and Eco-textiles Collaborative Innovation Center of Marine Biobased Fibers and Ecological Textiles Institute of Marine Biobased Materials Qingdao University Qingdao 266071 P. R. China; ^2^ School of Chemical Engineering The University of Queensland Brisbane 4072 Australia; ^3^ School of Materials Science and Engineering Yunnan Key Laboratory for Micro/Nano Materials and Technology Yunnan University Kunming Yunnan 650091 P. R. China; ^4^ Nanoscale Physics Research Laboratory School of Physics and Astronomy University of Birmingham Birmingham B15 2TT UK; ^5^ Hard X‐ray MicroAnalysis Beamline Facility Canadian Light Source Saskatoon S7N 0X4 Canada; ^6^ Analytical and Testing Centre South China University of Technology Guangzhou 510640 P. R. China; ^7^ Queensland Micro- and Nanotechnology Centre and School of Natural Sciences Griffith University Nathan Brisbane QLD 4111 Australia

**Keywords:** heterostructures, hydrogen evolution reaction, interfacial defects, O-refilling, P-vacancy, water splitting

## Abstract

Water electrolysis is a promising option for pure hydrogen production, but it is limited by the high cost. Developing superb and low‐cost electrocatalysts for hydrogen evolution reaction (HER) is critical for cost reduction. Heterostructures are demonstrated with excellent HER activities, but still inferior to commercial Pt/C. Herein, vacancy type of defects is engineered into the interface of CoP_3_/Ni_2_P heterostructure by a plasma strategy. The as‐synthesized defective CoP_3_/Ni_2_P exhibits lower overpotentials than Pt/C. Its specific activity at overpotential of 50 mV is ≈2‐fold and 1.7‐fold higher than that of Pt/C in acidic and alkaline media, respectively. For water electrocatalysis, its current density reaches 215 mA cm^−2^ at 2.0 V, even satisfying the target of practical industrial water splitting. Theoretical calculations indicate that the interfacial defects reconstruct the electronic structure and accelerate the charge transfer, facilitating the adsorption of reactant and lowering the energy barrier of water dissociation, thereby improving HER activities.

## Graphical abstract

Defective CoP_3_/Ni_2_P heterostructures exhibit ultralow *η*_10_ of 21 and 37 mV in acidic and alkaline conditions, respectively, outperforming those of Pt/C. More importantly, defective CoP_3_/Ni_2_P can satisfy the requirements of practical industrial water splitting, outperforming almost all the reported catalysts. Theoretical calculation verifies the critical role of interfacial defects on hydrogen evolution reaction (HER) activity of CoP_3_/Ni_2_P.

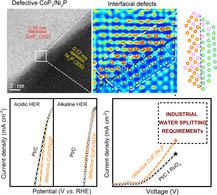

## 1 Introduction

Hydrogen has been extensively pursued as a clean and ideal energy carrier applied in many systems with little or no pollution, such as fuel cells, which generate power using a chemical reaction and produce only water/heat as by‐products.^[^ ^1^ ^]^ Water electrolysis, whereby water splits into hydrogen and oxygen by applying electrical energy, is a promising industrial process for almost pure hydrogen production.^[^ ^2^ ^]^ However, water electrolysis today satisfies only about 4% of the global hydrogen demand owing to its high cost.^[^ ^3^ ^]^ The cost is mainly contributed by two parts, e.g., the device system and electricity consumption.^[^ ^4^ ^]^ The current Pt‐based catalyst contributes the majority of the first part,^[^ ^5^ ^]^ while the insufficient activity of the catalyst (even Pt exhibits not enough low overpotential) is the reason for the latter part.^[^ ^6^ ^]^ Indeed, the latter part of cost for hydrogen production is more significant, which is about 3 times higher than the former.[^5^, ^7^] Therefore, it is essential to develop a sufficiently enough active catalyst (superior activity to Pt) for hydrogen evolution reaction (HER) with characteristics of non‐noble metal/s, enabling the wider application of hydrogen production by the water electrolysis.

Recently, earth‐abundant transition metal phosphides (TMPs, M=Co, Ni, Mo, Fe, Cu, etc.) have been regarded as the most promising candidates due to their remarkable HER activity and long‐term stability.^[^ ^6^, ^8^ ^]^ However, as a single HER electrocatalyst, all the reported TMPs are inferior to Pt‐based electrocatalysts, such as higher overpotentials at 10 mA cm^−2^ (*η*_10_ > 80 and 100 mV in acidic and alkaline conditions, respectively).^[^ ^9^ ^]^ It is known that the HER typically involves charge transfer between catalysts and adsorbed H^*^ and H_2_O. In this regard, proper adsorption strength of reactants and rapid charge transfer are beneficial for promoting a high‐performance HER electrocatalyst with fast reaction rate and low overpotential.^[^ ^10^ ^]^ Previous investigations have demonstrated that heterostructures, which are constructed by crystals with different electronic structures, can effectively reduce the *η*_10_ to ≈50 and 70 mV in acidic and alkaline conditions, respectively.^[^ ^11^ ^]^ This is mainly because the strong interfacial interaction can effectively modify the electronic structure of active sites, therefore optimizing the hydrogen adsorption strength.^[^ ^12^ ^]^ Nevertheless, those values are still higher than those of commercial Pt/C (*η*_10_ = ≈23 and 45 mV for acidic and alkaline solution, respectively). Thus, further electronic structure modifications on heterostructures are desirable, aiming to further accelerate the HER reaction rate. Very recently, a new concept of defect electrocatalysis was proposed with theoretical and experimental insights into the design of highly efficient electrocatalysts.^[^ ^13^ ^]^ Defects (anion/cation vacancy, lattice defects, dislocations, etc.) often give rise to an increase in free electron or hole concentration, resulting in an electron–hole asymmetry.^[^ ^14^ ^]^ This inspires us to create defects on the interface of a heterostructure to further modify the electronic structure, which may cause further electron delocalization and accelerate the charge transfer.

Herein, Ar‐plasma treatment was conducted on 2D CoP_3_/Ni_2_P nanosheet to generate defects at the interface of CoP_3_/Ni_2_P heterostructure (defective CoP_3_/Ni_2_P). Benefiting from the thin 2D structure, the Ar‐plasma is easy to impact upon the interface of CoP_3_/Ni_2_P. Thus, abundant phosphor vacancy defects (P‐vacancy) are also localized at the interface of heterostructures. Meanwhile, a part of those interfacial vacancy sites is refilled by the external O atoms to form O‐refilling defects. The defective CoP_3_/Ni_2_P achieves extremely low *η*_10_ of 21 and 37 mV in acidic and alkaline conditions, respectively, outperforming those of Pt/C (23 and 45 mV). The defective CoP_3_/Ni_2_P also exhibits outstanding oxygen evolution reaction (OER) activity with low *η*_10_ of 300 mV, even surpassing the commercial RuO_2_ (320 mV). Remarkably, as a bifunctional electrocatalyst for overall water splitting, the defective CoP_3_/Ni_2_P achieves 215 mA cm^−2^ at a voltage of 2.0 V, well fitting into the target of practical industrial water splitting and outperforming almost all the reported catalysts. Theoretical calculations reveal that the interfacial defects dramatically reconstruct the electronic structure and accelerate the charge transfer, thus optimizing the absorption of H* and lowering the energy barrier of water dissociation.

## 2 Results and Discussion

**Figure 1:**
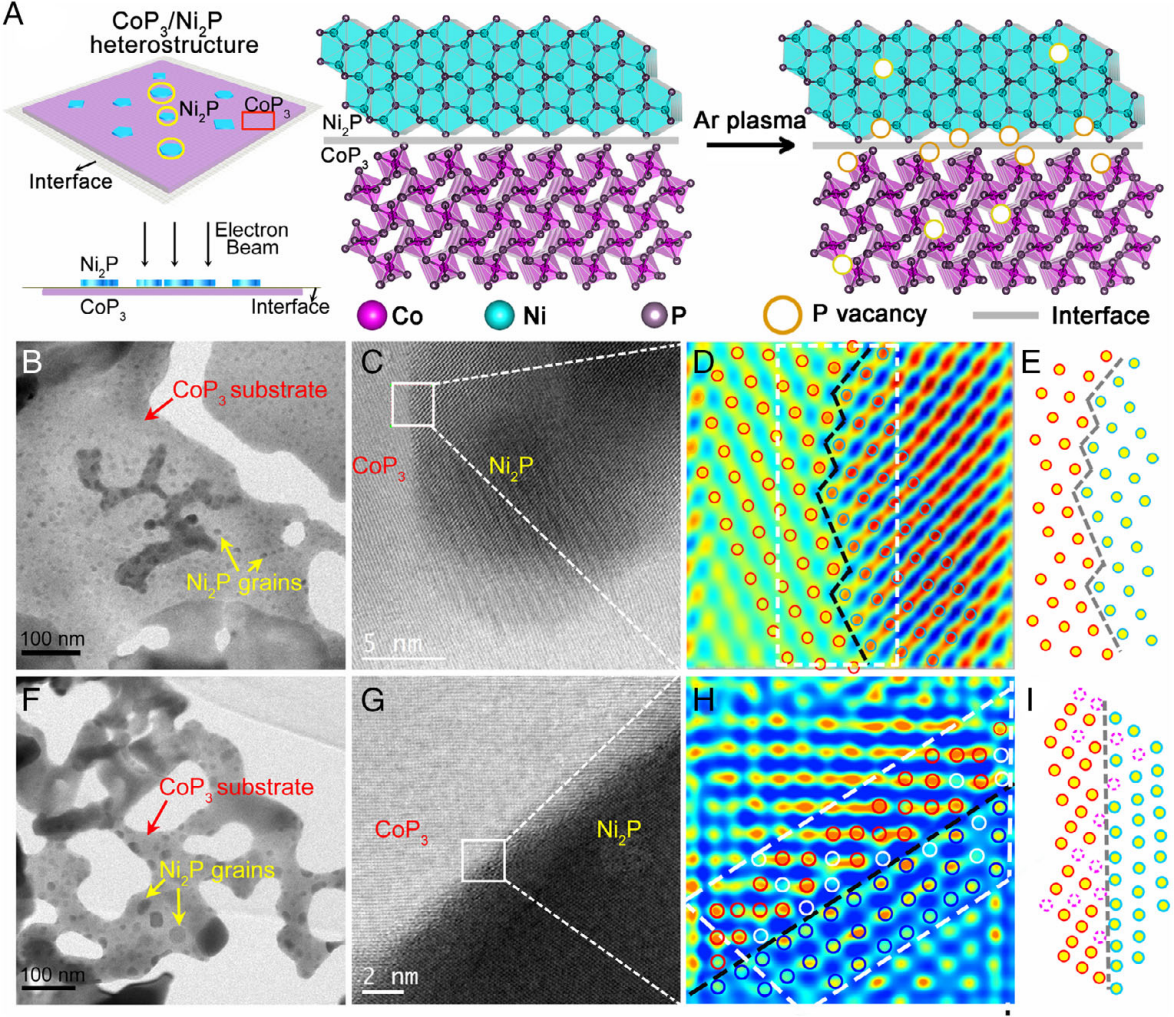
A) Illustration of preparation of defective CoP_3_/Ni_2_P. B) TEM image, C) HAADF‐STEM image, D) filtered image, and E) the model of interface at CoP_3_/Ni_2_P. F) TEM image, G) HAADF‐STEM image, H) filtered image, and I) the model of interface at defective CoP_3_/Ni_2_P.

The preparation procedure of defective CoP_3_/Ni_2_P is shown in **Figure** **1**A. First, the pristine 2D CoP_3_/Ni_2_P nanosheets with thickness of ≈4 nm were fabricated (Figure S1–S3, Supporting Information). The CoP_3_/Ni_2_P heterostructure combines firmly along well‐matched phase interface between (33$\bar{6}\bar{2}$) plane of Ni_2_P and ($\bar{6}\bar{2}$111) plane of CoP_3_, which has been verified in our previous report.^[^ ^15^ ^]^ Then, the pristine 2D CoP_3_/Ni_2_P heterostructures were subjected to Ar microwave plasma treatment at 100 W with the reactor pressure keeping at 0.5–1.0 Pa for different times (15, 30, and 45 min), obtaining defective CoP_3_/Ni_2_P (CoP_3_/Ni_2_P–*t*, *t* is the treating time). Due to the ultrathin 2D characteristic, Ar‐plasma can penetrate through the nanosheets easily, generating abundant defects at the interface as well as surface.

The transmission electron microscopy (TEM) and high‐resolution TEM (HRTEM) images clearly show that for both CoP_3_/Ni_2_P and defective CoP_3_/Ni_2_P, dark Ni_2_P grains are deposited on the bright CoP_3_ substrate (Figure 1B,F, S4, and S5, Supporting Information). The high‐angle annular dark‐field scanning transmission electron microscopy (HAADF‐STEM) was performed to observe their interfacial difference. The HAADF‐STEM image of CoP_3_/Ni_2_P clearly shows a distinct boundary between the lattice of CoP_3_ and Ni_2_P (Figure 1C). Apart from the boundary marked with black dotted line, the corresponding filtered image shows consecutive lattice fringes without defects at the interface (Figure 1D). A perfect CoP_3_/Ni_2_P interface model without defects is shown in Figure 1E. In contrast, the boundary of defective CoP_3_/Ni_2_P between those two crystals becomes ambiguous (Figure 1G). Intermittent lattice fringes are observed in the corresponding filtered image (Figure 1H). Thus, for defective CoP_3_/Ni_2_P, a defective model with abundant defects at the interface is shown in Figure 1I. Those defects are mainly induced by the escape of P atoms owing to the Ar‐plasma. As a consequence, abundant defects are engineered into the interface via plasma treatment of CoP_3_/Ni_2_P heterostructure.

To reveal the chemical conversions from CoP_3_/Ni_2_P to defective CoP_3_/Ni_2_P, X‐ray photoelectron spectroscopy (XPS) was conducted. The XPS spectra of CoP_3_/Ni_2_P and CoP_3_/Ni_2_P–*t* exhibit the same peak locations (Figure S6–S9, Supporting Information). However, it is found that the ratio of metal–P/metal–O content becomes lower and decreases with the extension of plasma treatment (Table S1, Supporting Information). For example, the Co–P/Co–O content ratios of CoP_3_/Ni_2_P and CoP_3_/Ni_2_P‐30 calculated from Co 2p spectra are 4.50 and 3.34, respectively, suggesting that plasma treatment leads to a decrease in Co–P content and increase in Co–O. The Ni–P/Ni–O content ratio calculated from Ni 2p spectra decreases from 18.40% of CoP_3_/Ni_2_P to 13.84% of CoP_3_/Ni_2_P‐30, also suggesting a decrease in Ni–P and increase in Ni–O. The Co/Ni–P/P–O content ratio calculated from P 2p spectra decreases from 1.72% (CoP_3_/Ni_2_P) to 1.30% (CoP_3_/Ni_2_P‐30), consistent with the fitting results of Co and Ni XPS spectra. Apparently, all these results suggest that P‐vacancy and O‐refilling sites are generated via Ar‐plasma treatment.

**Figure 2:**
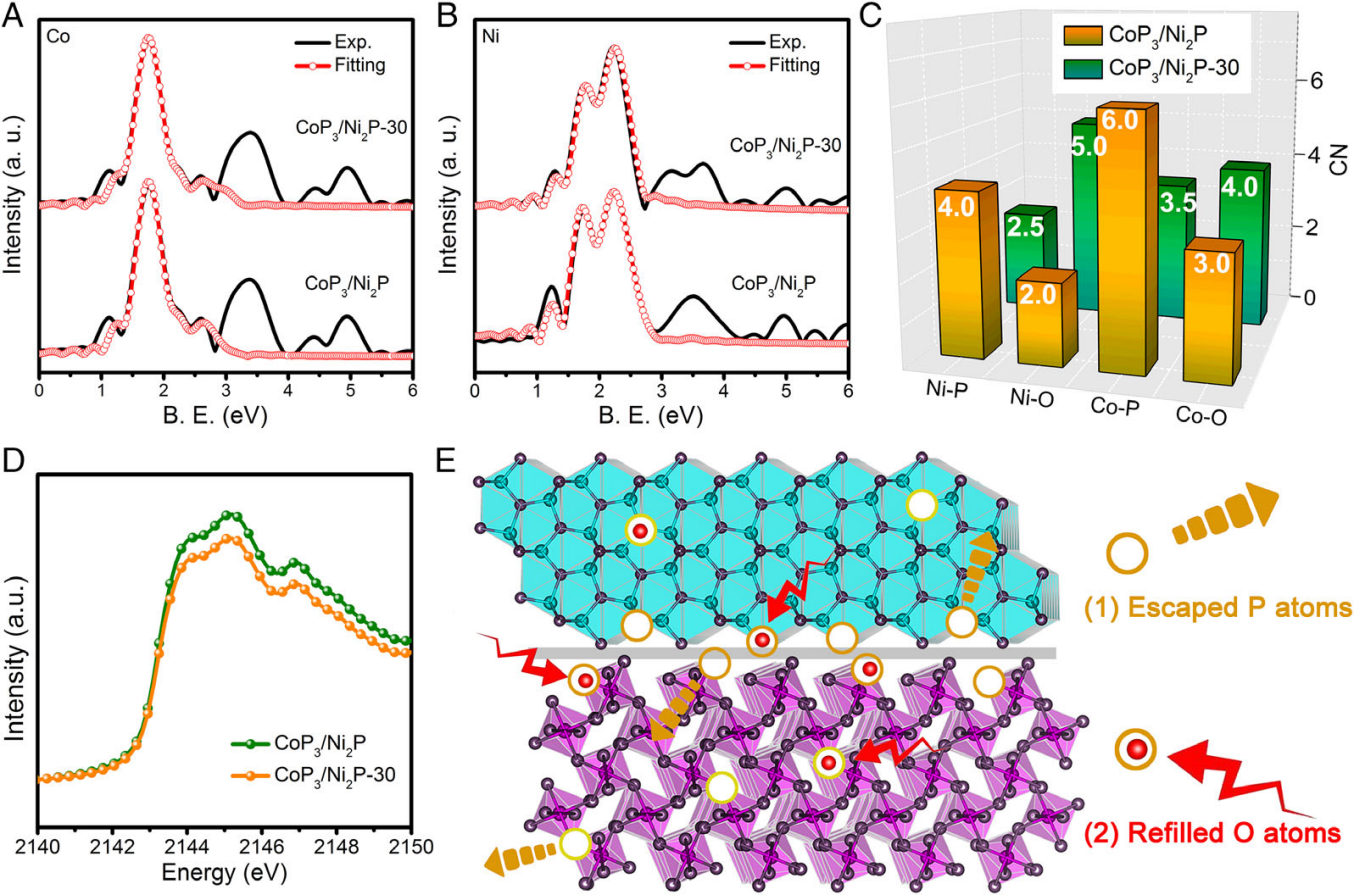
A,B) Co and Ni K‐edge EXAFS fitting curves of CoP_3_/Ni_2_P and CoP_3_/Ni_2_P‐30, respectively. C) The CNs of metal–O and metal–P paths for CoP_3_/Ni_2_P and CoP_3_/Ni_2_P‐30. D) P K‐edge XAS spectra of CoP_3_/Ni_2_P and CoP_3_/Ni_2_P‐30. E) The structural illustration of the defective CoP_3_/Ni_2_P.

To finely investigate the structure and chemical environment, X‐ray absorption near‐edge structure (XANES) and extended X‐ray absorption fine structure (EXAFS) measurements were conducted. Obviously, compared with CoP_3_/Ni_2_P, the XANES and EXAFS of Co and Ni K‐edge for CoP_3_/Ni_2_P‐30 display a similar spectrum shape with different amplitude (Figure S10, Supporting Information), indicating their different local atomic arrangements.^[^ ^16^ ^]^ EXAFS fitting analysis of Co and Ni K‐edge was performed to compare the coordination environments of CoP_3_/Ni_2_P and CoP_3_/Ni_2_P‐30 (**Figure** **2**A,B and Table S2–S3, Supporting Information). As shown in Figure 2C, the coordination numbers (CNs) of Co–P and Ni–P for CoP_3_/Ni_2_P‐30 are 3.5 and 2.5, respectively, which are much lower than those of CoP_3_/Ni_2_P (6.0 for Co–P, 4.0 for Ni–P). This means the creation of a large number of P‐vacancy sites in plasma treatment. Meanwhile, compared with CoP_3_/Ni_2_P, the CNs of Co–O and Ni–O for CoP_3_/Ni_2_P‐30 increase from 3.0 and 2.0 to 4.0 and 5.0, respectively, indicating that a partial of the P‐vacancies is refilled by external O atoms. This is consistent with the XPS results. In addition, compared with CoP_3_/Ni_2_P, the P K‐edge XANES intensity of CoP_3_/Ni_2_P‐30 is relatively reduced (Figure 2D), indicating a lower P concentration in CoP_3_/Ni_2_P‐30, further demonstrating the escape of P atoms after Ar‐plasma treatment. Based on the XPS and EXAFS analysis combined with the aforementioned STEM images, the structure of defective CoP_3_/Ni_2_P can be deduced and illustrated, as shown in Figure 2E. Surface and interfacial defects of P‐vacancy defects (formed by the escaped P atoms) and O‐refilling defects (formed by occupying a partial of those P‐vacancies sites with O atoms) are generated in defective CoP_3_/Ni_2_P.

**Figure 3:**
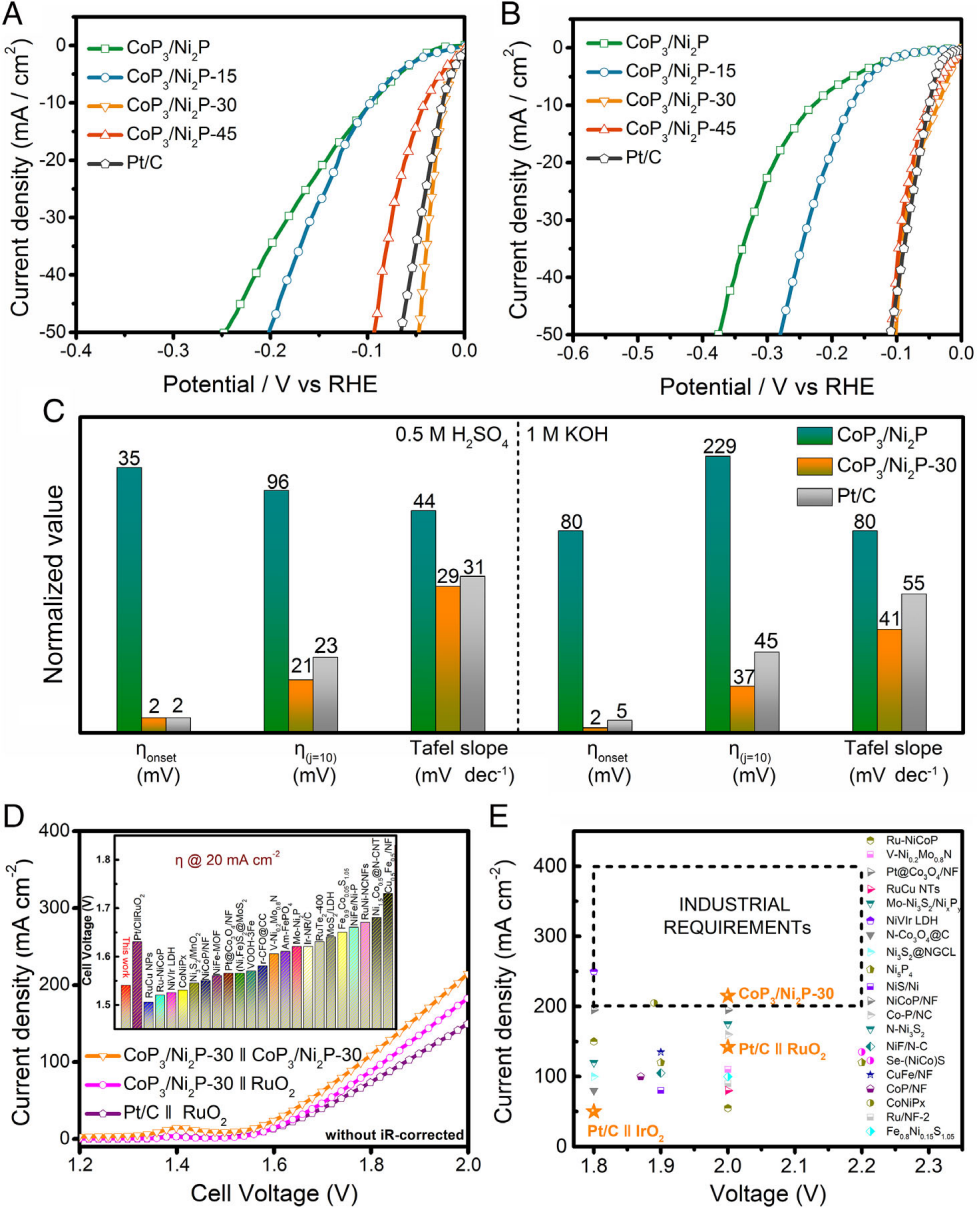
A,B) Polarization curves in 0.5 m H_2_SO_4_ and 1 m KOH, respectively. C) Comparison of *η*_onset_, *η*_10_ and Tafel slope of CoP_3_/Ni_2_P, CoP_3_/Ni_2_P‐30, and commercial Pt/C. The heights of the CoP_3_/Ni_2_P‐30 bars were normalized, and the heights of the CoP_3_/Ni_2_P and commercial Pt/C bars were altered accordingly. D) Polarization curves of CoP_3_/Ni_2_P‐30 ‖ CoP_3_/Ni_2_P‐30, CoP_3_/Ni_2_P‐30 ‖ RuO_2_, and Pt/C ‖ RuO_2_ coupled catalysts in a two‐electrode configuration for overall water splitting in 1.0 m KOH without *iR*‐correction (both loaded into Ni foam at a loading of 2 mg cm^−2^). The inset is the required voltage to obtain a current density of 20 mA cm^−2^ for CoP_3_/Ni_2_P‐30 ‖ CoP_3_/Ni_2_P‐30 and other state‐of‐the‐art noble metal free catalysts. E) The current densities of CoP_3_/Ni_2_P‐30 ‖ CoP_3_/Ni_2_P‐30 and other state‐of‐the‐art noble metal free catalysts for overall water splitting in 1.0 m KOH in the voltage range of 1.8–2.4 V.

The interfacial defects can efficiently boost HER performance in both acidic and alkaline electrolytes. As shown in **Figure** **3**A,B and S11, Supporting Information, the HER activity of the pristine CoP_3_/Ni_2_P in 0.5 m H_2_SO_4_ and 1 m KOH is much inferior to that of the commercial Pt/C in aspects of both onset overpotential (*η*_onset_, overpotential at 1 mA cm^−2^) and *η*_10_. However, the HER performance can be greatly improved with plasma treatment. Particularly, with the extension of plasma treatment time to 30 min (CoP_3_/Ni_2_P‐30), the sample achieves the best activity in both 0.5 m H_2_SO_4_ and 1.0 M KOH solutions. As shown in Figure 3C, apart from a very small *η*_onset_ of 2 mV, the CoP_3_/Ni_2_P‐30 only requires an extremely small overpotential of 21 mV to drive a current density of 10 mA cm^−2^ in 0.5 m H_2_SO_4_, which is much better than those of Pt/C (23 mV) and pristine CoP_3_/Ni_2_P (96 mV). Similarly, in 1 m KOH, the CoP_3_/Ni_2_P‐30 exhibits an *η*_onset_ of 2 mV and *η*_10_ of only 37 mV, which also largely outperform those of commercial Pt/C (5 and 45 mV, respectively) and CoP_3_/Ni_2_P (80 and 229 mV, respectively). Further extension of the treatment time (CoP_3_/Ni_2_P‐45) will cause slight performance decay. This might stem from the destroyed crystallinity and decreased electrical conductivity induced by excessive defects (Figure S3 and S12, Supporting Information). Notably, CoP_3_/Ni_2_P‐30 realizes a complete nonprecious metal electrocatalyst surpassing Pt/C in both acidic and alkaline media for HER electrocatalysis. In addition, the HER performance of the CoP_3_/Ni_2_P‐30 was also tested under 1 m PBS (Figure S13, Supporting Information), but unfortunately, it exhibits inferior catalytic activity to Pt/C under neutral conditions.

Tafel plots are used to investigate the reaction kinetics and mechanism of HER. A Tafel slope of 29 mV dec^−1^ is measured for CoP_3_/Ni_2_P‐30 in 0.5 m H_2_SO_4_ (Figure 3C and S14, Supporting Information), which is lower than that of Pt/C (31 mV dec^−1^), CoP_3_/Ni_2_P (44 mV dec^−1^), CoP_3_/Ni_2_P‐15 (42 mV dec^−1^), and CoP_3_/Ni_2_P‐45 (36 mV dec^−1^). In 1.0 m KOH, CoP_3_/Ni_2_P‐30 exhibits a Tafel slope of 41 mV dec^−1^, also smaller than that of Pt/C (55 mV dec^−1^), CoP_3_/Ni_2_P (80 mV dec^−1^), CoP_3_/Ni_2_P‐15 (69 mV dec^−1^), and CoP_3_/Ni_2_P‐45 (44 mV dec^−1^). As a smaller Tafel slope indicates a faster reaction kinetic, CoP_3_/Ni_2_P‐30 possesses the fastest kinetic rate. The charge transfer resistance calculated from electrical impedance spectroscopy (EIS) also reveals the fast charge transfer in CoP_3_/Ni_2_P‐30 during the HER processes in both acidic and alkaline (Figure S12, Supporting Information). Experimentally, Tafel slope is also used to elucidate the possible HER mechanisms. Normally, the reaction will undergo a Volmer–Tafel mechanism for a Tafel slope of ≈30 mV dec^−1^, or a Volmer–Heyrovsky mechanism for a Tafel slope of ≈40 mV dec^−1^.^[^ ^17^ ^]^ Consequently, CoP_3_/Ni_2_P‐30 and Pt/C undergo a Volmer–Tafel mechanism and Volmer–Heyrovsky mechanism in acid and alkaline media, respectively, and CoP_3_/Ni_2_P undergoes a Volmer–Heyrovsky mechanism in both acid and alkaline media.

Apart from the common geometric area activity, specific activities normalized to the effective active surface area of electrocatalysts also should be investigated to further quantify the intrinsic activity of electrocatalysts. Electrochemically active surface area (ECSA) offers a measurement of the surface area directly participated in the electrochemical reaction. The ECSA of those samples was estimated by double‐layer capacitance (*C*_dl_) that tested using a cyclic voltammetry method (Figure S15 and Table S4, Supporting Information). The normalized specific activities are shown in Figure S16A,B, Supporting Information. Apparently, the CoP_3_/Ni_2_P‐30 requires the smallest overpotentials to reach the current density of 0.1 mA m_ECSA_^−2^ in both acidic and alkaline solutions (17, 32 mV), which are much less than those of Pt/C (30, 60 mV), CoP_3_/Ni_2_P (152, 302 mV), CoP_3_/Ni_2_P‐15 (124, 199 mV), and CoP_3_/Ni_2_P‐45 (44, 55 mV), indicating the highest intrinsic catalytic activity of CoP_3_/Ni_2_P‐30 (Figure S16C, Supporting Information). It is worth noting that, as shown in Figure S16D, Supporting Information, under a small overpotential of 50 mV, CoP_3_/Ni_2_P‐30 delivers current densities of 0.65 and 0.16 mA cm_ECSA_^−2^ in acidic and alkaline solutions, respectively, which are about 3 and 2.7 times that of Pt/C (0.22, 0.06). The positive effect of defects on inherent activity was also demonstrated by the turnover frequency (TOF). As shown in Figure S17, Supporting Information, defective CoP_3_/Ni_2_P exhibits higher TOF values than those of pristine CoP_3_/Ni_2_P. Especially, the TOF values of CoP_3_/Ni_2_P‐30 are about 20 and 33 times higher than those of CoP_3_/Ni_2_P in 0.5 m H_2_SO_4_ and 1 m KOH, respectively. Apart from high catalytic activity, CoP_3_/Ni_2_P‐30 also exhibits remarkable catalytic and structure stability (Figure S18–S20, Supporting Information). After electrocatalysis for 30 h, the CoP_3_/Ni_2_P‐30 maintains high current retentions of 94.7% and 84.6% in acidic and alkaline, respectively.

As water electrolysis includes not only HER on cathode but also OER on anode, the OER activities of defective CoP_3_/Ni_2_P have been also investigated in 1.0 m KOH (Figure S21, Supporting Information). Apparently, to drive a current density of 10 mA cm^−2^, the overpotential of CoP_3_/Ni_2_P‐30 (300 mV) is smaller than that of commercial RuO_2_ (320 mV) and CoP_3_/Ni_2_P (350 mV), indicating a high OER activity. The outstanding OER activity of defective CoP_3_/Ni_2_P could largely facilitate the overall water splitting ability. To further assess the actual performance in water electrolysis, a two‐electrode electrolyzer was assembled with CoP_3_/Ni_2_P‐30 as both anode and cathode (CoP_3_/Ni_2_P‐30 ‖ CoP_3_/Ni_2_P‐30). The water splitting activity of the cell is measured in 1.0 m KOH. For comparison, the CoP_3_/Ni_2_P‐30 paired with RuO_2_ (CoP_3_/Ni_2_P‐30 ‖ RuO_2_) and commercial 20% Pt/C paired with RuO_2_ loaded on Ni foam (Pt/C ‖ RuO_2_) were assembled and tested under the same conditions. From Figure 3D and inset, it can be observed that the CoP_3_/Ni_2_P‐30 ‖ CoP_3_/Ni_2_P‐30 requires a low voltage of 1.55 V to obtain a current density of 20 mA cm^−2^, which obviously outperforms that of CoP_3_/Ni_2_P‐30 ‖ RuO_2_ (1.63 V), Pt/C ‖ RuO_2_ (1.63 V), and most of the state‐of‐the‐art noble metal free catalysts (commonly 1.6–1.7 V). Importantly, for practical industrial alkaline water splitting, it is required a current density of 200–400 mA cm^−2^ in a voltage of 1.8–2.4 V.[^4^, ^18^] The polarization curves show that the current density of CoP_3_/Ni_2_P‐30 ‖ CoP_3_/Ni_2_P‐30 is 215 mA cm^−2^ at a voltage of 2.0 V, apparently meeting the requirements of practical industrial water splitting. Obviously, the water splitting activity of CoP_3_/Ni_2_P‐30 ‖ CoP_3_/Ni_2_P‐30 is far beyond that of CoP_3_/Ni_2_P‐30 ‖ RuO_2_ (183 mA cm^−2^ at a voltage of 2.0 V), Pt/C ‖ RuO_2_ (150 mA cm^−2^ at a voltage of 2.0 V), and most of the nonprecious metal‐based catalysts (Figure 3E).^[^ ^19^ ^]^ Thus, at the voltage of 2 V, compared with Pt/C ‖ RuO_2_, the CoP_3_/Ni_2_P‐30 ‖ CoP_3_/Ni_2_P‐30 could produce much more H_2_ in the same time, and reduce the electricity consumption at least by one‐third to produce 1 kg H_2_. The H_2_ and O_2_ production of CoP_3_/Ni_2_P‐30 ‖ CoP_3_/Ni_2_P‐30 was measured quantitatively by gas chromatography. As shown in Figure S22, Supporting Information, the molar ratio of H_2_ and O_2_ is very close to 2:1, and the measured amounts are well matched with the calculated values, indicating a high Faradic efficiency of almost 100% for CoP_3_/Ni_2_P‐30. In addition, the catalytic stability of CoP_3_/Ni_2_P‐30 ‖ CoP_3_/Ni_2_P‐30 was assessed by electrolysis at a static voltage of 1.8 V. Phenomenally, the evolution of H_2_ and O_2_ gas bubbles could be clearly observed (Movie S1, Supporting Information). The initial current density achieves as high as 100 mA cm^−2^, and after 30 000 s, the current density retention is 87% (Figure S23, Supporting Information). In contrast, the Pt/C ‖ RuO_2_ displays an initial current density of 74 mA cm^−2^ with the current density retention of 82% after 30 000 s. Therefore, the defective CoP_3_/Ni_2_P catalyst is more likely an ideal candidate over Pt for scalable electrosplitting of water in alkaline media. However, as for the overall water splitting in 0.5 m H_2_SO_4_ (Figure S24, Supporting Information), CoP_3_/Ni_2_P‐30 ‖ CoP_3_/Ni_2_P‐30 exhibits inferior activity to CoP_3_/Ni_2_P‐30 ‖ RuO_2_ and Pt/C ‖ RuO_2_, attributing to its poor OER activity in acidic media. A selection of other highly efficient OER catalysts in acid media is desirable.

**Figure 4:**
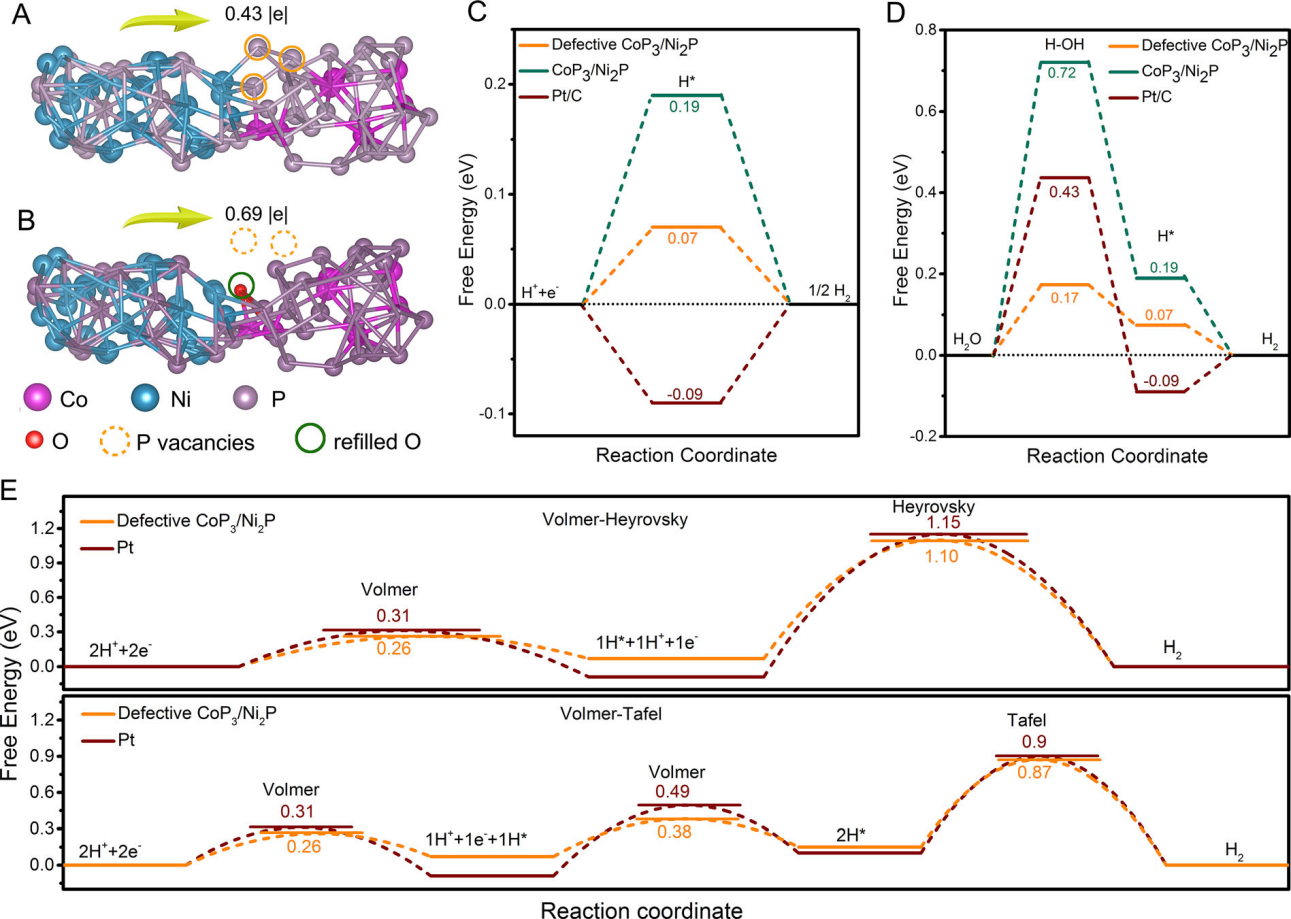
Optimized structural representations of A) CoP_3_/Ni_2_P and B) defective CoP_3_/Ni_2_P. Free energy diagrams of the HER pathways for CoP_3_/Ni_2_P, defective CoP_3_/Ni_2_P, and Pt under C) acidic and D) alkaline conditions, respectively. E) The kinetic energy barrier profiles of and Volmer–Heyrovsky routes (up) and Volmer–Tafel routes (bottom) for defective CoP_3_/Ni_2_P and Pt.

To shed more light on the interface defects on the catalytic hydrogen evolution, theoretical calculations were conducted using the density functional theory (DFT). We built pristine CoP_3_/Ni_2_P model with (33$\bar{6}$ $\bar{2}$) Ni_2_P/($\bar{1}$11) CoP_3_ as the interface, and based on the CoP_3_/Ni_2_P model, defective CoP_3_/Ni_2_P model was built through removing part of the surface interfacial P atoms to form P‐vacancy defects, and then refilling some of those P‐vacancy sites with O atoms to form O‐refilling defects (**Figure** **4**A,B). Apparently, the introduction of P‐vacancy and O‐refilling defects brings distortion to neighboring atoms. The defect effect on electronic structures is first investigated by the charge density differences (Figure S25A,B, Supporting Information). Obviously, for both CoP_3_/Ni_2_P and defective CoP_3_/Ni_2_P, charge transfer occurs at the interface. Much more significant charge accumulation can be observed on the defective CoP_3_/Ni_2_P, indicating that the interfacial defects could increase charge density, so as to facilitate the adsorption of reactants of H* and H_2_O.^[^ ^20^ ^]^ Bader charge was used to analyze the electron loss/gain of each interfacial atom. Compared with pristine CoP_3_/N_2_P, the Bader charge numbers of interfacial atoms in defective CoP_3_/Ni_2_P have changed more greatly, demonstrating the redistribution of electrons (Figure S25C,D, Supporting Information). Based on Bader analysis, the defective CoP_3_/Ni_2_P exhibits a higher charge transfer of 0.69 |e| than CoP_3_/Ni_2_P of 0.43 |e|, demonstrating that interfacial defects accelerate the charge transfer. The enhanced charge transfer will lead to a fast reaction rate and further to an enhanced catalytic activity.^[^ ^21^ ^]^

To further demonstrate the positive effect of interfacial defects on HER activity, the free energy diagrams in acidic and alkaline media were calculated, respectively. The active sites and corresponding Gibbs free energies of CoP_3_/Ni_2_P and defective CoP_3_/Ni_2_P models are shown in Figure S26 and Table S5–S6, Supporting Information. In acidic electrolyte, the overall HER pathway is composed of three states, including an initial state H^+^, adsorbed H*, and H_2_ desorption (Figure 4C).^[^ ^22^ ^]^ A Gibbs free energy of H* absorbed on catalyst (Δ*G*_H*_) that approximates to 0 eV is highly desirable for a superior HER catalyst.^[^ ^23^ ^]^ The Δ*G*_H*_ of CoP_3_/Ni_2_P (0.19 eV) is quite high, indicating the weak interaction between H* and active sites, which could result in an inferior reaction efficiency. For defective CoP_3_/Ni_2_P, the significantly increased charge density helps to strongly absorb the H* and largely decrease the Δ*G*_H*_ to 0.07 eV, thus prominently improving the HER activity. The Δ*G*_H*_ of defective CoP_3_/Ni_2_P is even more promising than that of Pt (−0.09 eV) for HER, as the ultralow adsorption energy could hinder hydrogen desorption.^[^ ^24^ ^]^ Therefore, the acidic HER activity of defective CoP_3_/Ni_2_P outperforms that of CoP_3_/Ni_2_P and Pt, which is consistent with the experimental results. Compared with acidic HER, the mechanism of alkaline HER is different, which includes an initial H_2_O dissociation to H* intermediates and generation of H_2_.^[^ ^25^ ^]^ The H_2_O dissociation is the rate‐determining step for HER activity in alkaline electrolyte due to its high energy barrier. Unfortunately, the CoP_3_/Ni_2_P exhibits an unfavorable activated H_2_O adsorption energy (Δ*G*_H2O_) of 0.72 eV (Figure 4D), and such a high energy barrier hinders the water dissociation into H*, resulting in a sluggish alkaline HER kinetics. In contrast, the defective CoP_3_/Ni_2_P significantly reduces the Δ*G*_H2O_ to 0.17 eV, which is even smaller than that of Pt (0.43 eV), benefiting the water dissociation. Thus, defective CoP_3_/Ni_2_P exhibits a much better alkaline HER activity than CoP_3_/Ni_2_P and Pt/C, which is also consistent with the experimental results.

To further investigate the detailed HER pathways of CoP_3_/Ni_2_P, defective CoP_3_/Ni_2_P, and Pt, the kinetic energy barriers of the transition states in the different reaction pathways are calculated. According to the Tafel slope analysis (Figure 3C), CoP_3_/Ni_2_P‐30 and Pt/C undergo a Volmer–Tafel and Volmer–Heyrovsky pathway in 0.5 m H_2_SO_4_ and 1.0 m KOH, respectively, while CoP_3_/Ni_2_P undergoes a Volmer–Heyrovsky pathway in both solutions. Therefore, kinetic energy barrier profiles of both Volmer–Heyrovsky and Volmer–Tafel reaction pathways on defective CoP_3_/Ni_2_P and Pt are plotted (Figure 4E), and only that of Volmer–Heyrovsky reaction pathway on CoP_3_/Ni_2_P is calculated (Figure S27, Supporting Information). In the Volmer–Heyrovsky pathway, for CoP_3_/Ni_2_P, the kinetic energy barrier for the rate‐limited Heyrovsky step reaches up to 1.25 eV, which may hinder the production of H_2_. But for defective CoP_3_/Ni_2_P, this value can be significantly decreased to 1.10 eV, even smaller than that of Pt (1.15 eV). Meanwhile, in the Volmer–Tafel pathway diagram, the kinetic energy barrier for the rate‐limited Tafel step of defective CoP_3_/Ni_2_P (0.87 eV) is also lower than that of Pt (0.90 eV). These results indicate that interfacial defects of CoP_3_/Ni_2_P can remarkably reduce the kinetic energy barrier, leading to the superior acidic and alkaline HER activities of defective CoP_3_/Ni_2_P.

## 3 Conclusion

In summary, P‐vacancy and O‐refilling defects are successfully engineered into the interface of CoP_3_/Ni_2_P heterostructure via Ar‐plasma treatment. The as‐prepared defective CoP_3_/Ni_2_P largely lowers the *η*_10_ to 21 and 37 mV in acidic and alkaline conditions, respectively, outperforming those of Pt/C. For actual water electrolysis, the defective CoP_3_/Ni_2_P reaches a high current density of 215 mA cm^−2^ at the cell voltage of 2.0 V, meeting the requirements of industrial water splitting (200–400 mA cm^−2^ in the cell voltage of 1.8–2.4 V). DFT calculations demonstrate that the interfacial defects redistribute electrons, accelerating the charge transfer from 0.43 to 0.69 |e|. The optimized electronic structure facilitates the adsorption of H* (reducing Δ*G*_H*_) and promotes the water dissociation (lowering Δ*G*_H–OH_), consequently improving the acidic and alkaline HER activities. This work verifies the critical role of interfacial defects on HER activity of CoP_3_/Ni_2_P both experimentally and theoretically, and may pave a new way for the design of highly active catalysts.

## 4 Experimental Section

4.1

4.1.1

##### Synthesis of Pristine 2D CoP_3_/Ni_2_P Heterostructure

In a typical procedure, 0.0349 g Co(NO_3_)_2_ 6H_2_O, 0.0285 g NiCl_2_ 6H_2_O, and 0.0720 g urea were dissolved in deionized water (80 mL). Then, the solution was transferred to a 100 mL Teflon‐lined stainless steel autoclave and heated to 150 °C for 3 h. After cooling down, the CoNi layered double hydroxide (LDH) precursors were collected. Subsequently, the CoNi LDH (100 mg) and NaH_2_PO_2_ (1.0 g) in a porcelain boat were placed at two separate positions of the tube furnace, and heated at 650 °C for 1 h in Ar atmosphere, obtaining pristine 2D CoP_3_/Ni_2_P heterostructure.

##### Synthesis of Defective CoP_3_/Ni_2_P Heterostructure

The pristine 2D CoP_3_/Ni_2_P (60 mg) was spread on a quartz boat and inserted into a plasma reactor. Subsequently, the reactor was pumped down in Ar atmosphere (keep a flowing rate of 4.5 mL min^−1^) until the pressure decreased to 0.5–1.0 Pa. The pristine 2D CoP_3_/Ni_2_P was treated with Ar‐plasma at the power of 100 W for 15, 30, and 45 min, respectively, obtaining defective CoP_3_/Ni_2_P (denoted as CoP_3_/Ni_2_P–*t*).

## Conflict of Interest

The authors declare no conflict of interest.

## Data Availability Statement

Data openly available in a public repository that issues datasets with DOIs.

## Supporting information

Supplementary Material

Supplementary Material
