# Supplementary material for: Beyond Platinum: Defects Abundant CoP3/Ni2P Heterostructure for Hydrogen Evolution Electrocatalysis
Source: Small Sci. 2021 Mar 6;1(4):2000027. doi: 10.1002/smsc.202000027 (PMC11935819; doi:10.1002/smsc.202000027)
Supplement: Supplementary file 1 — Supplementary Material [file SMSC-1-2000027-s002.pdf]

Supplementary data

Beyond Platinum: Defects Abundant CoP_3/Ni_2P Heterostructure for Hydrogen Evolution Electrocatalysis

Lijie Zhang[£], Linzhou Zhuang[£], Hongli Liu, Rongsheng Cai, Longzhou Zhang, Ning Chen, Xianfeng Yang, Zhonghua Zhu, Dongjiang Yang* and Xiangdong Yao*

[£]L. J. Zhang and L. Z. Zhuang contributed equally to this work.

*Corresponding authors: D.J. Yang, d.yang@qdu.edu.cn; X.D. Yao, x.yao@griffith.edu.au.

This PDF file includes:

Details for materials characterizations, the calculation of specific activity and DFT calculations

Figure S1 to S27

Table S1 to S6

Electrochemical measurement.

All the electrochemical tests were performed in a conventional three-electrode system at an electrochemical station (Biologic VMP2/Z multichannel potentiostat), using Ag/AgCl (3 M NaCl) electrode as the reference electrode, graphitic carbon rod as the counter electrode and glassy carbon (GC) electrode as the working electrode. Thin film electrodes were prepared by sonicating 5.0 mg active catalyst for 30 minutes in 0.5 mL ethanol with 50 µL 5 wt% Nafion solution. 5 µL of this suspension was drop-cast onto a glassy carbon disk electrode (4 mm diameter, 0.126 cm^2 area) and left to dry under a glass jar. The typical catalyst loading was 0.36 mg cm^{-2}. The stability was tested using Ti mesh (1×1 cm^2) loaded catalyst as the working electrode to facilitate the diffusion of generated bubbles. The catalyst loading on Ti mesh was 0.36 mg cm^{-2}. The polarization curves are iR corrected with the solution resistance, where it is 9 and 13 Ω for 0.5 M H_2SO_4 and 1 M KOH, respectively. To estimate the faradic efficiency of full water splitting, a CoP_3/Ni_2P-30-based two-electrode electrolyzer was assembled and assessed at an initial current density of about 25 mA cm^{-2} for 160 min. The quantities of the generated H_2 and O_2 were determined every 20 min by using an GC-2014C (SHIMADZU) gas chromatograph.

Materials Characterizations

The morphologies of the samples were characterized by field emission scanning electron microscopy (FESEM; SU8020). Transmission electron microscopy were collected from FEI Tecnai G20 and probe-corrected JEOL ARM200F with

acceleration voltages of 120 and 80 kV, respectively. The chemical composition was investigated by X-ray photoelectron spectroscopy (XPS) using an ESCALab250 electron spectrometer (Thermo Scientific Corporation) with mono-chromatic 150 W Al Kα radiation. The phase structures were characterized with X-ray diffraction (XRD, DX2700, China) operating with Cu Kα radiation (l=1.5418 Å) at a scan rate (2θ) of 1° min^{-1} with the accelerating voltage of 40 kV. For X-ray absorption near edge structure (XANES) and extended X-ray absorption fine structure (EXAFS), the data collected at hard X-ray microanalysis (HXMA) beamline (BL), Canada Light Source (CLS). CLS storage ring operation at 250 mA mode, the beamline superconducting wiggler source was operated at1.7 T. The monochromatic X-ray beam was produced by using a double crystal Si (111) monochromator with its second crystal detuned by 50% at the end of the XAFS scan to reduce the high harmonic components in the X-ray beam. The beamline was configured in its focused mode with Rh mirrors (collimating and focusing mirrors) in the X-ray beam path. The experiment was performed in florescence mode by a 32 elements Ge detector, equipped with solar slits, arsenic filter (3 absorption length), and 4 layers of Al foil. The mono initial energy calibration was performed by using selenium elemental foil from EXAFS materials. During the experiment each individual XAFS scan was further calibrated by using the data in-step collected from the same Se foil, which was located between the two ion chamber detectors downstream of the sample. All three ion chamber detectors was filled with 100% He.

The calculation of specific activity

Specific activity is the activity (current obtained for the linear sweep voltammetry test) normalized to the electrochemical active surface area (ECSA). In this work, we use electrochemical double layer capacitances (C_{dl}) to characterize electrochemical active surface area (ECSA). The C_{dl} was measured by cyclic voltammograms in a potential region of 0.05 V to 0.15 V *vs.* RHE, where there is no Faradic current. The C_{dl} is estimated by plotting the ΔJ (J_a-J_c) at 0.1 V *vs*. RHE against the scan rates, where the slope is twice C_{dl}. The ECSA of 1 cm^2 is represented by a special capacitance value 0.2 mF cm^{-2}. ECSA and ECSA current density (j_{ECSA}) were calculated by the following equation

Here, the ECSA was estimated from the electrochemical double layer capacitance (C_{dl}). To study the C_{dl} of CoP_3/Ni_2P and CoP_3/Ni_2P-*t*, we conducted the CV cycles at different scan rates in the range of potential from 0.05 V to 0.15 V vs RHE, where there is no Faradic current.

According to C_{dl} is constant, it can be calculated as:

$$C_{dl} = \frac{Q}{U} = \frac{dQ/dt}{dU/dt} = \frac{j}{r}$$

Q is the quantity of electric charge per unit area,

U is the voltage, *j* is the current density and *r* is the scan rate.

The calculations of turnover frequency (TOF)

The TOF is calculated by the following Equation,

$$TOF = \frac{j * S}{2 * F * N}$$

The *F* is Faraday constant (96485 C mol^{-1}), the *j* is measured current density at an overpotential of 50 mV, S is the surface area of working electrode (0.126 cm^2), and the number of 2 means 2 electrons mol^{-1} of H_2, and N is the moles of Co and Ni atoms on

electrode. In this work, the molar ratio of Co and Ni is 1:1, and the mass loading of catalyst on the working electrode is 0.045 mg. Thus, the moles of Co and Ni atoms on working electrode are 1.5×10^{-4} mmol.

DFT calculations

The density functional theory (DFT) calculations were carried out using the Vienna ab Initio Simulation package (VASP).[2,3] The ion–electron interactions were described by the projector plane wave (PAW) approach. Electron exchange–correlations were represented by the functional of Perdew, Burke and Ernzerhof (PBE) of generalized gradient approximation (GGA).[4] To ensure the convergence for total energy, all calculations were performed using a plane-wave cutoff energy of 400eV with Fermi-level smearing of 0.1 eV and Monkhorst-Pack grid (3×3×1) was used for k-point sampling. Besides, the convergence threshold of energy and forces were set to be 1×10^{-5} eV and 0.02 eV/Å, respectively.

HER in acidic media

The overall acidic HER pathway includes two steps: adsorption of hydrogen on the catalyst (H^*) from initial state H^++e^-+*), and release of molecular hydrogen ($1/2H_2+*$), where * denotes the catalyst.

The Gibbs free-energy of the adsorption of atomic hydrogen (ΔEH ΔE_H) is obtained by Eq (1)

$$\Delta G^0_{H*} = \Delta E_H + \Delta E_{ZPE} - T\Delta S_H \quad (1)$$

Where ΔE_{ZPE} , and ΔS_H are respectively the difference in zero point energy, and entropy between hydrogen adsorption and hydrogen in the gas phase. The contributions from the catalysts to both ΔE_{ZPE} and ΔS_H are small and are neglected. Therefore, ΔE_{ZPE}^{nH} is obtained by Eq (2)

$$\Delta E_{ZPE} = E_{ZPE}^{nH} - E_{ZPE}^{(n-1)H} - \frac{1}{2} E_{ZPE}^{H_2} \quad (2)$$

Where, ΔE_{ZPE}^{nH} is the zero point energy of n-adsorbed hydrogens on the catalyst, without the contribution of catalyst. The ΔS_H is obtained by Eq (3)

$$\Delta S_H \cong -\frac{1}{2} S_{H_2}^{0} \quad (3)$$

And S_{H2}^{0} is the entropy of H_2 gas at the standard condition. Therefore, Eq (4) can be rewritten as Eq (4):

$$\Delta G_{H*}^{0} = \Delta E_H + 0.24\ eV \quad (4)$$

ΔE_H is the differential hydrogen adsorption energy, which is defined by Eq (5):

$$\Delta E_H = E_{nH^*} - E_{(n-1)H^*} - \frac{1}{2} E_{H_2} \quad (5)$$

Where * donates the catalyst. E_{nH*}, $E_{(n-1)H*}$ and E_{H_2} represent total energies of catalyst plus n adsorbed hydrogen atoms, total energies of catalyst plus n-1 adsorbed hydrogen atoms, and gas H_2, respectively.

The theoretical HER model is based on the assumption that the total energies of $H^+(aq) + e^-$ and $\frac{1}{2} H_2(g)$ are equal under standard conditions (pH = 0). Therefore, HER is usually performed under condition of pH=0 in DFT calculations.

HER in alkaline media

The key reaction steps in alkaline HER:

$H_2O + e^- + cat \rightarrow H^*\text{-}cat + OH^-$ (6)

$2H^*\text{-}cat \rightarrow H_2$ (7)

$H^*\text{-}cat + H_2O + e^- \rightarrow cat + OH^- + H_2$ (8)

The free energy for (6) and (8) should be the same at equilibrium potential of HER. Under this assumption, one can avoid computation of the exact free energy of OH^- in solutions by using computational hydrogen electrode.[5] Herein, four main stage are considered: initial state, activated water adsorption, H* intermediates formation, H_2 formation.[6] The free energies (at the reduction potentials U_0=0 V vs RHE) are calculated as:

$$G_0 = G_{cat} + G_{H_2O}$$

$$G_1 = G_{cat-(H-OH)}$$

$$G_2 = G_{cat-H*} + G_{OH^-}$$

$$G_3 = G_{cat} + G_{OH^-} + \frac{1}{2} G_{H_2}$$

$$G_3 = G_0$$

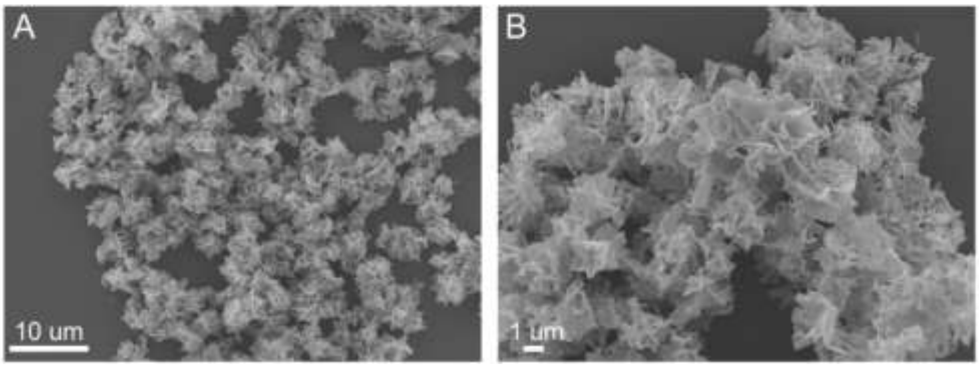

Fig. S1│(A) FESEM image of the as-synthesized CoP_3/Ni_2P and (B) its amplified SEM image.

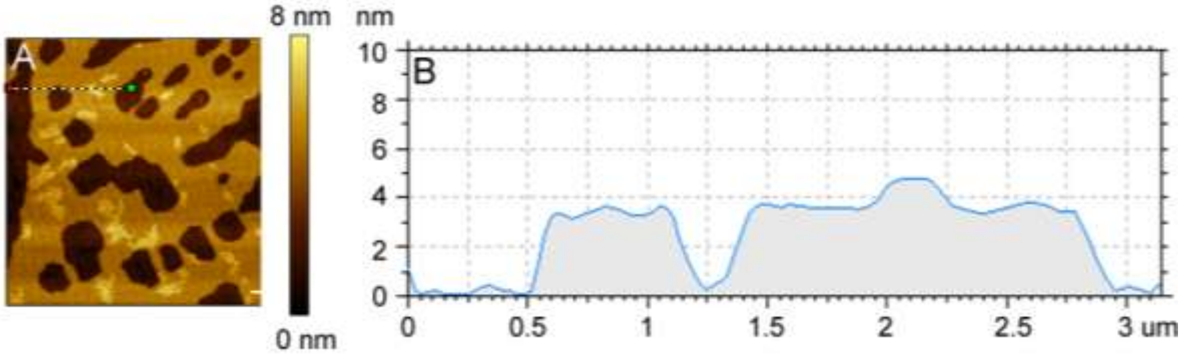

Fig. S2│(A) AFM height image and (B) corresponding height *vs*. position curve (dotted line in A) of a CoP_3/Ni_2P nanosheet.

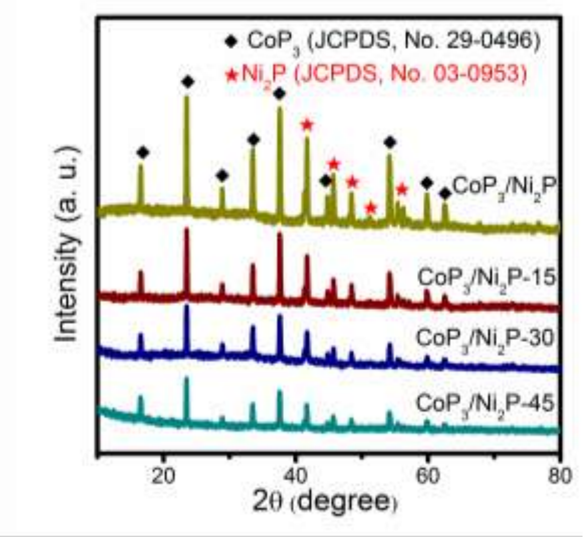

Fig. S3│XRD pattern of CoP_3/Ni_2P and defective CoP_3/Ni_2P with different Ar-plasma time (CoP_3/Ni_2P-*t*, *t* is the Ar-plasma time).

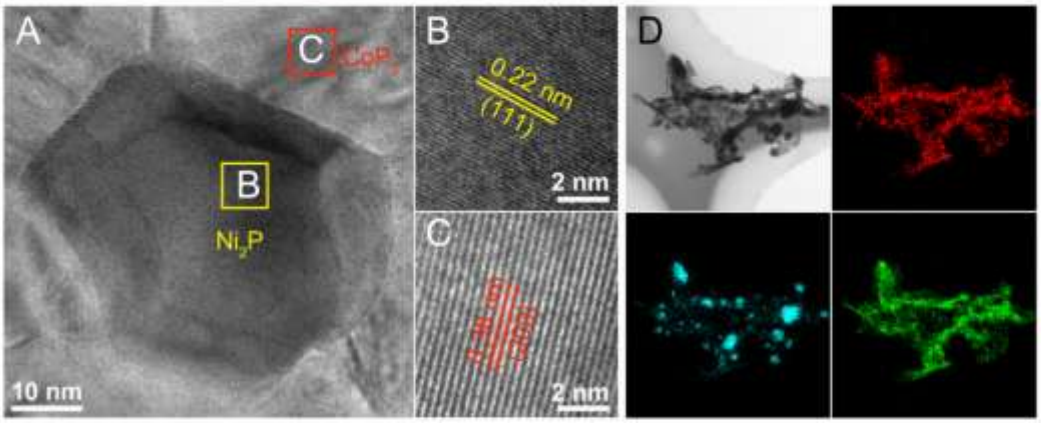

Fig. S4│TEM image (A) and (B-C) corresponding HRTEM image of Ni_2P grain (marked in yellow square) and CoP_3 grain ((marked in red square)) in (A), respectively, and EDX mappings of Co, Ni and P atoms (D) for CoP_3/Ni_2P.

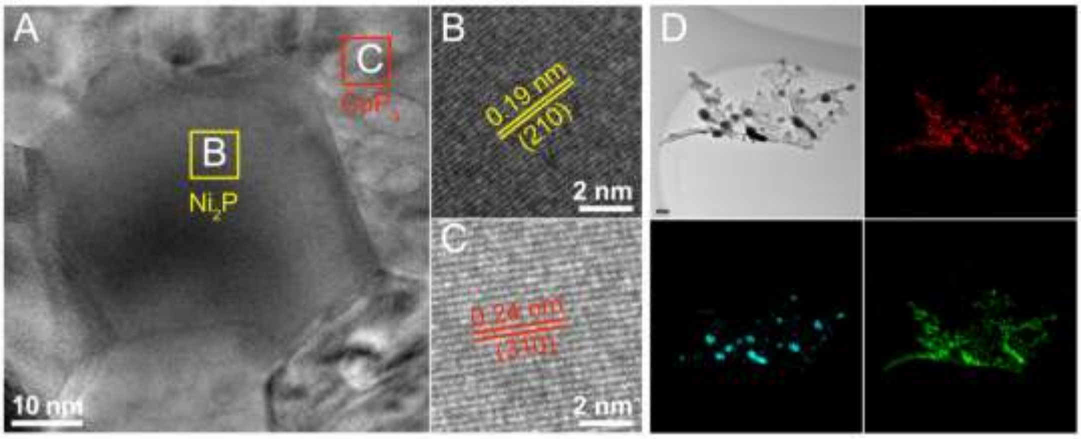

Fig. S5│TEM image (A) and (B-C) corresponding HRTEM image of Ni_2P grain (marked in yellow square) and CoP_3 grain ((marked in red square)) in (A), respectively, and EDX mappings of Co, Ni and P atoms (D) for defective CoP_3/Ni_2P.

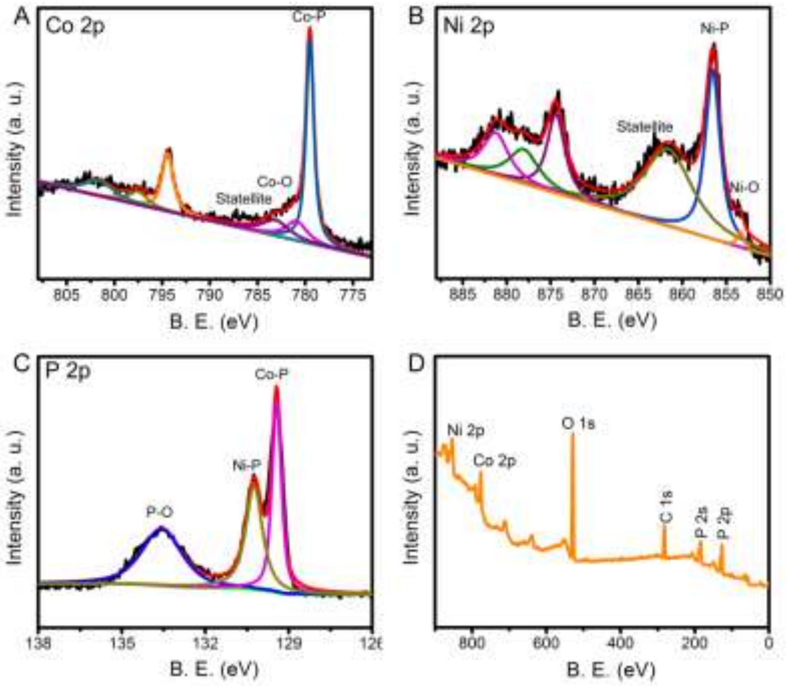

Fig. S6│High resolution XPS of Co 2p (A), Ni 2p (B) and P 2p (C), and full scan spectrum (D) of CoP_3/Ni_2P.

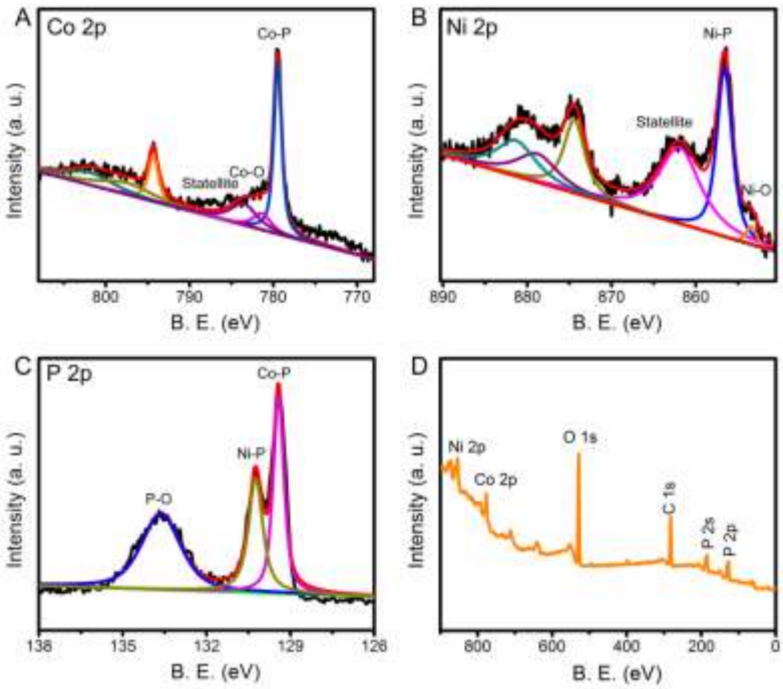

Fig. S7│High resolution XPS of Co 2p (A), Ni 2p (B) and P 2p (C), and full scan spectrum (D) of CoP_3/Ni_2P-15.

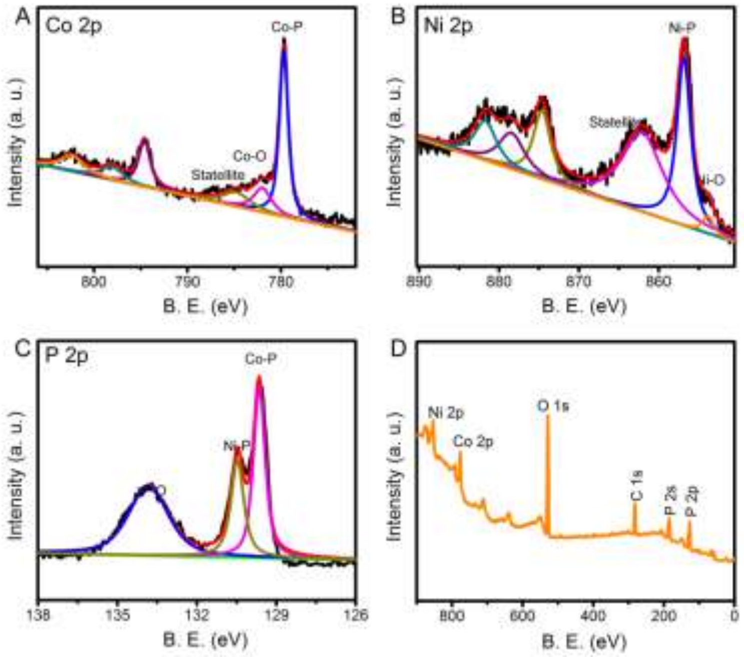

Fig. S8│High resolution XPS of Co 2p (A), Ni 2p (B) and P 2p (C), and full scan spectrum (D) of CoP_3/Ni_2P-30.

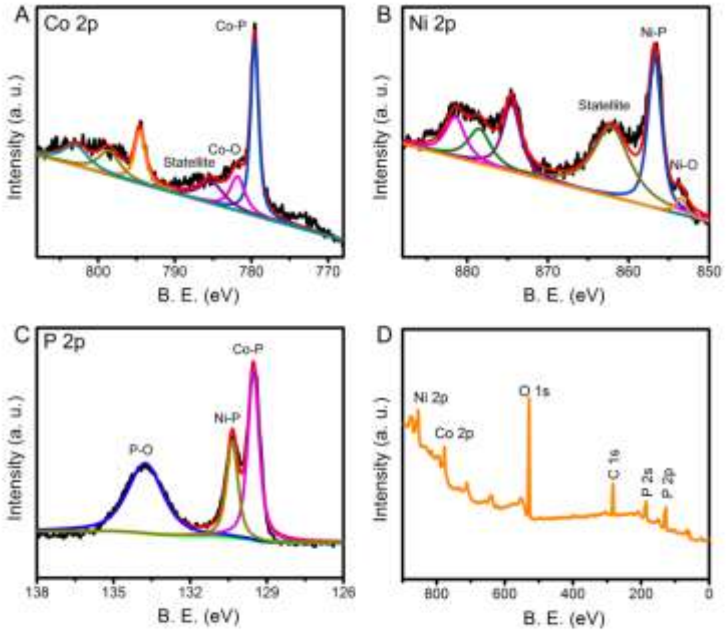

Fig. S9| High resolution XPS of Co 2p (A), Ni 2p (B) and P 2p (C), and full scan spectrum (D) of CoP_3/Ni_2P-45.

For Co 2p spectra, the peaks located at 779.5 and 781.6 eV correspond to Co-P and Co-O bonding, respectively.[7,8] For Ni 2p spectra, the peaks located at 856.6 and 853.5 eV are ascribed to Ni-P and Ni-O, respectively.[9,10] As for the P 2p spectra, two peaks at 129.5 ($2p_{3/2}$) and 130.4 eV ($2p_{1/2}$) correspond to the binding energies of CoP_3 and Ni_2P, the peak located at 133.5 eV corresponds to the oxidized P species.[11-13]

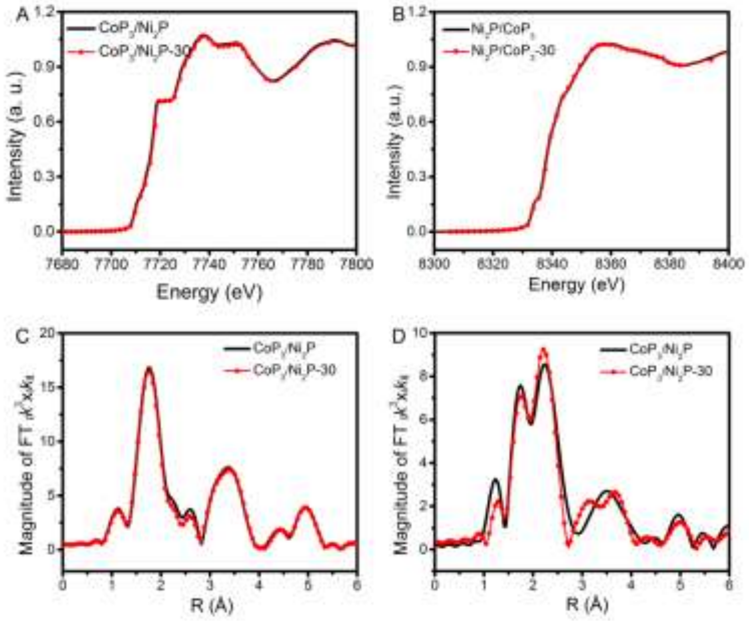

Fig. S10│Co K-edge (A) and Ni K-edge (B) XANES spectra of CoP_3/Ni_2P and CoP_3/Ni_2P-30, respectively. Fourier transforms of the experimental Co K-edge (C) and Ni K-edge (D) EXAFS spectra of CoP_3/Ni_2P and CoP_3/Ni_2P-30, respectively.

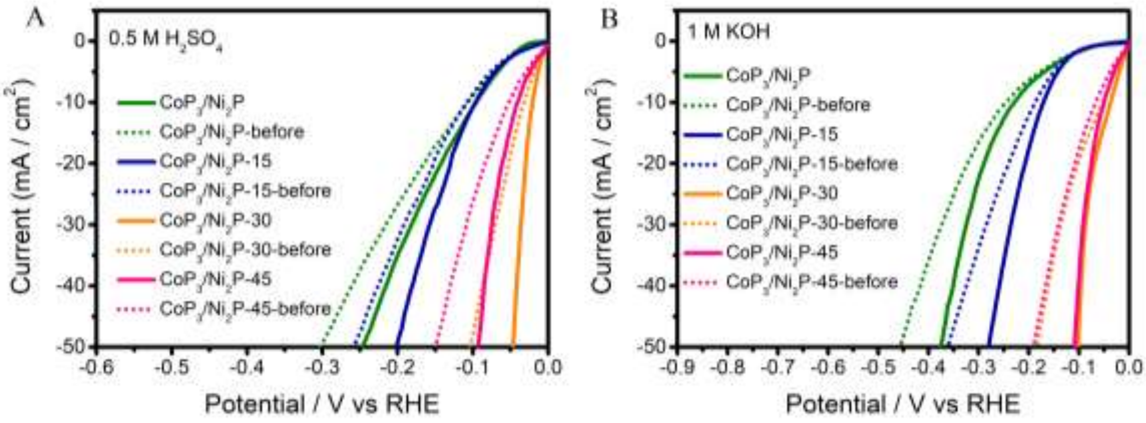

Fig. S11│ Polarization curves for CoP_3/Ni_2P and CoP_3/Ni_2P-*t* before and after iR corrected in 0.5 M H_2SO_4 (A) and 1 M KOH (B).

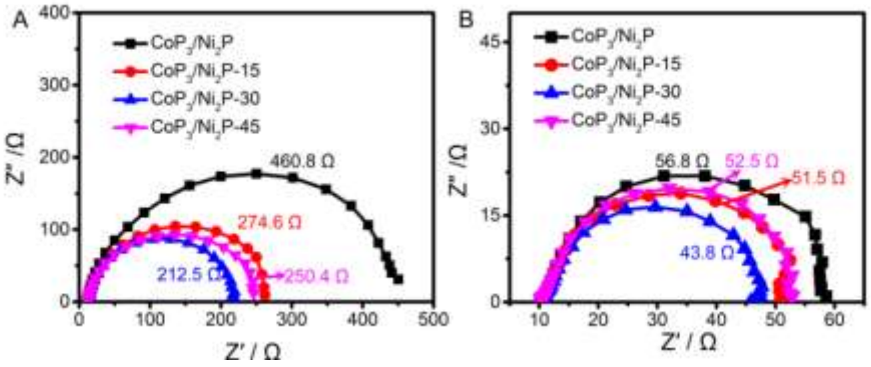

Fig. S12│Electrochemical impedance spectra (EIS) of CoP_3/Ni_2P and CoP_3/Ni_2P-*t*. in 0.5 M H_2SO_4 (A) and 1.0 M KOH (B), respectively.

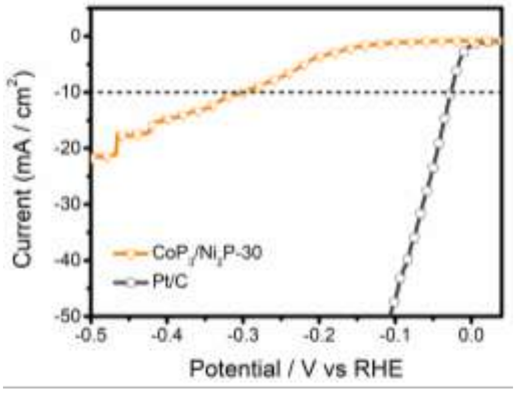

Fig. S13│Polarization curves of Pt/C and CoP_3/Ni_2P-30 in 1 M PBS.

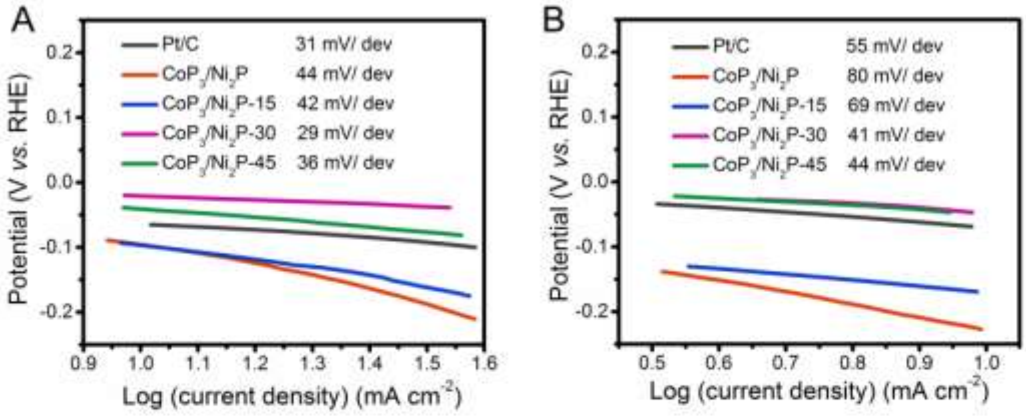

Fig. S14│ Tafel plts of Pt/C, CoP_3/Ni_2P and defective CoP_3/Ni_2P in 0.5 M H_2SO_4 (A) and 1 M KOH (B), respectively.

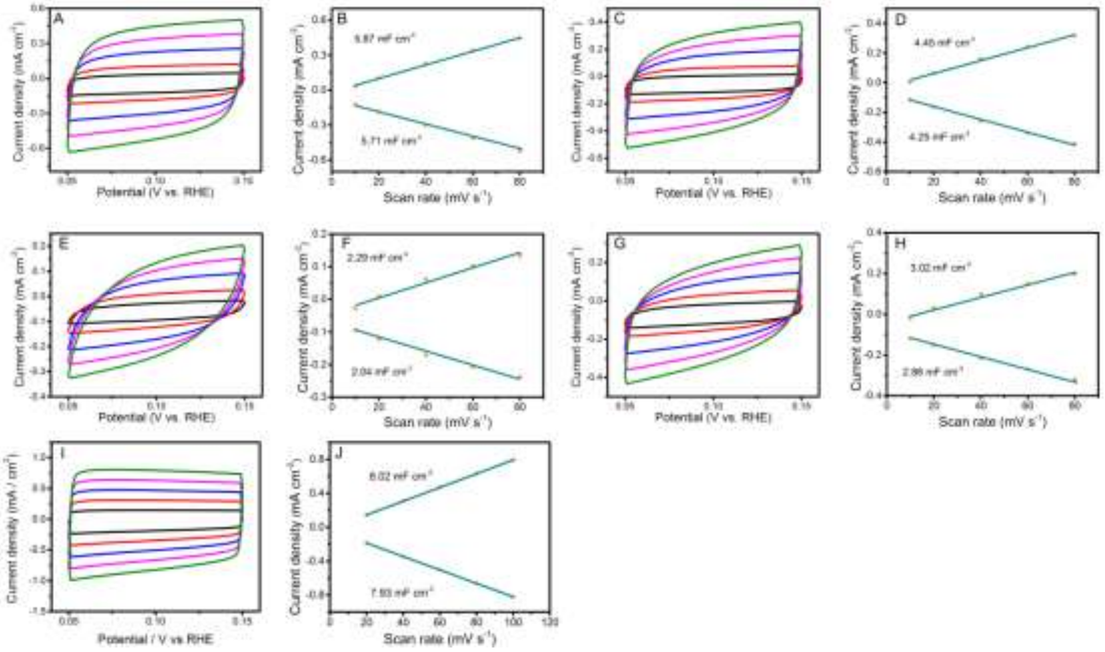

Fig. S15│CV curves at various scan rates of CoP_3/Ni_2P (A), CoP_3/Ni_2P-15 (C), CoP_3/Ni_2P-30 (E), CoP_3/Ni_2P-45 (G) and Pt/C (I). The plots of the current density versus the scan rate for CoP_3/Ni_2P (B), CoP_3/Ni_2P-15 (D), CoP_3/Ni_2P-30 (F), CoP_3/Ni_2P-45 (H) and Pt/C (J).

Due to that the ECSA of 1 cm^2 is represented by a special capacitance value 0.2 mF cm^{-2}, the ECSA of CoP_3/Ni_2P, CoP_3/Ni_2P-15, CoP_3/Ni_2P-30 and CoP_3/Ni_2P-45 are 28.95, 21.75, 10.83 and 14.7 cm^{-2}, respectively.

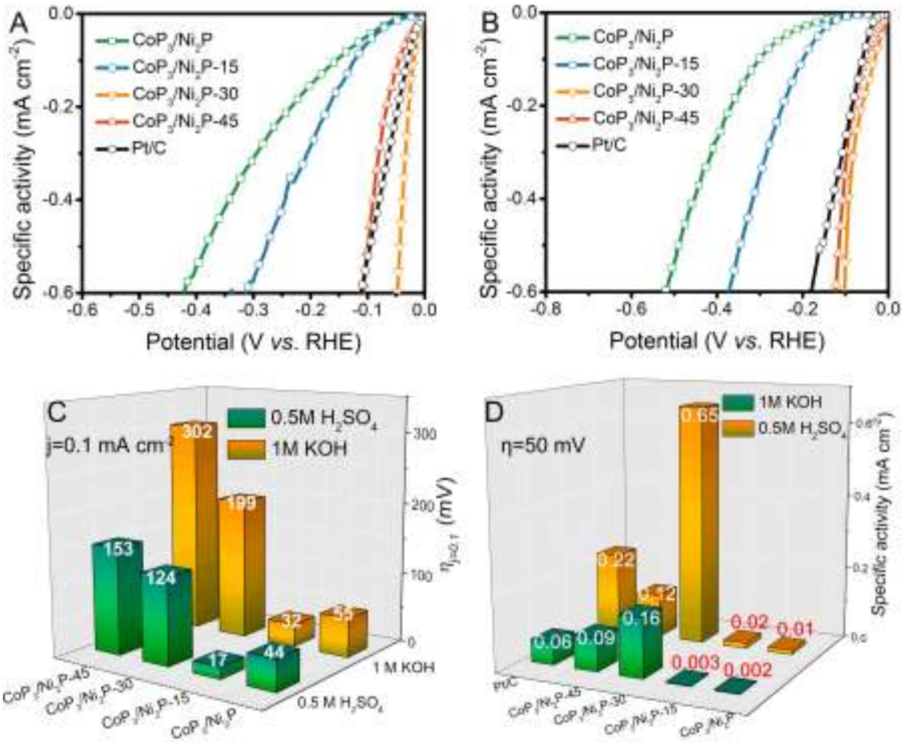

Fig. S16│ LSVs normalized to the electrochemically active surface area (ECSA) in 0.5 M H_2SO_4 (A) and 1 M KOH (B), respectively. (C) The corresponding overpotentials to drive a current density of 0.1 mA cm^{-2}. (D) The corresponding specific activities at the overpotential of 50 mV.

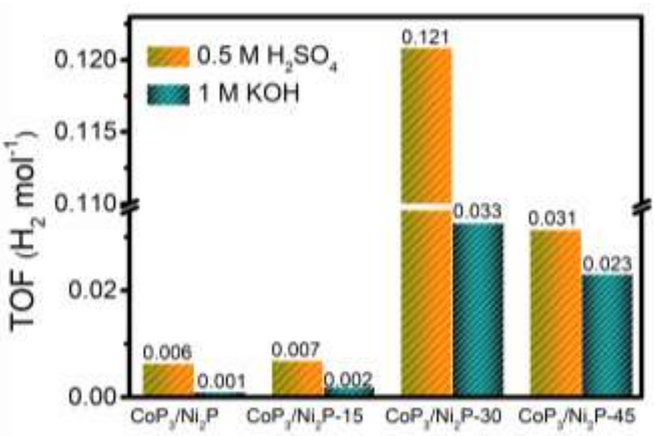

Figure S17│ Calculated TOF values of CoP_3/Ni_2P and defect CoP_3/Ni_2P at the overpotential of 50 mV.

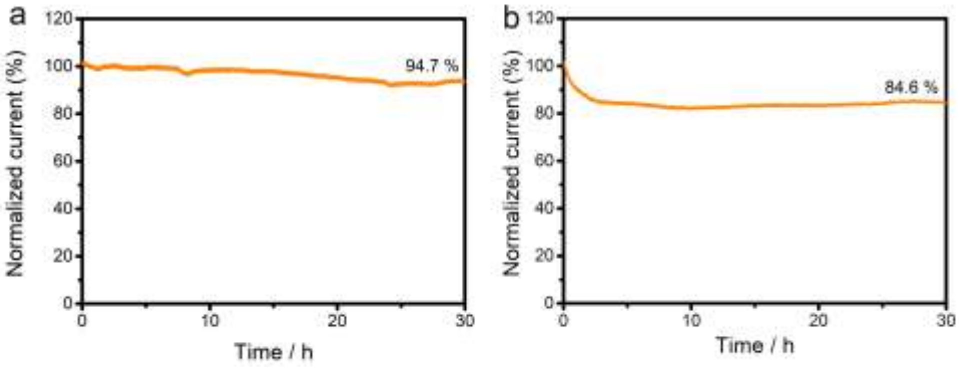

Fig. S18│ The long-term stability tests of CoP_3/Ni_2P-30 at 10 mA cm^{-2} in acidic (A) and alkaline (B) conditions.

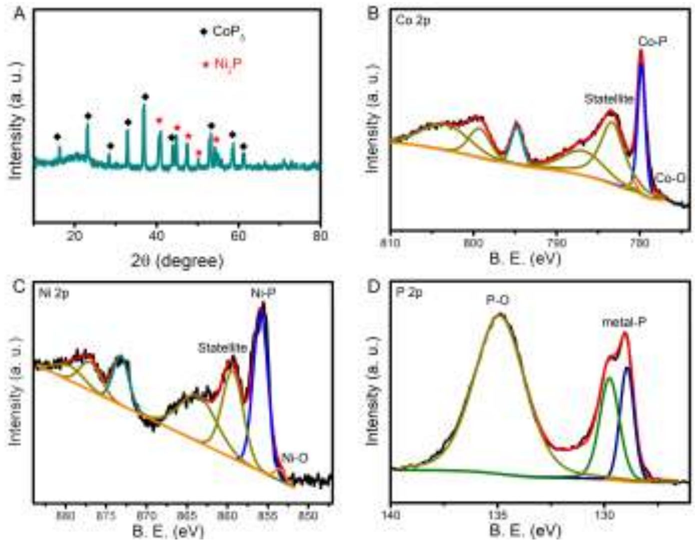

Fig. S19│ (A) XRD pattern, high resolution XPS spectra of Co 2p (B), Ni 2p (C), and P 2p (D) for CoP_3/Ni_2P-30 after HER test in 1 M KOH.

After HER test, the diffraction peaks corresponding to CoP_3 and Ni_2P are clearly observed in the XRD patterns, implying a well preservation of CoP_3/Ni_2P. The peak positions for XPS after catalysis remain the same with the initial sample, indicating the stable chemical states of CoP_3/Ni_2P.

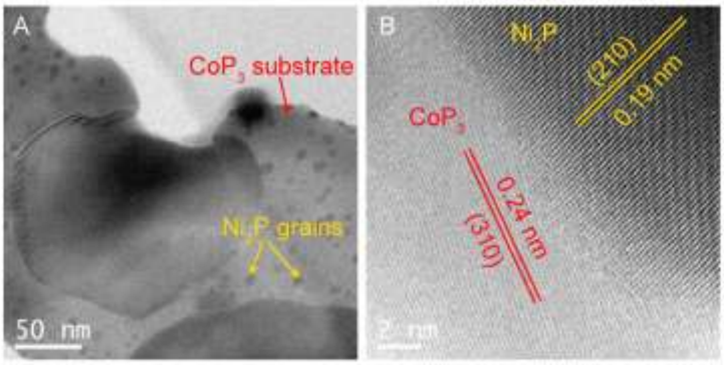

Fig. S20│ (A) TEM and (B) HRTEM images of CoP_3/Ni_2P-30 after HER test in 1 M KOH.

As observed from the TEM and HRTEM images of CoP_3/Ni_2P-30 after catalysis, dark Ni_2P grains are deposited on the bright CoP_3 substrate, consistent with the initial samples, indicating the structural stability of CoP_3/Ni_2P.

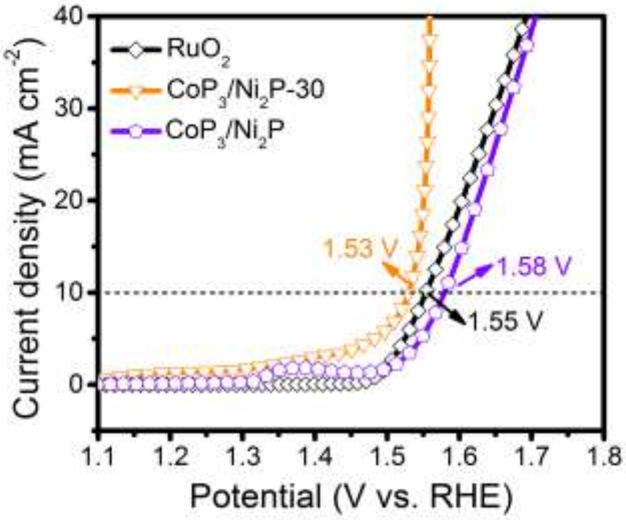

Fig. S21│ The electrochemical OER activities of CoP_3/Ni_2P-30, CoP_3/Ni_2P and RuO_2 in 1 M KOH.

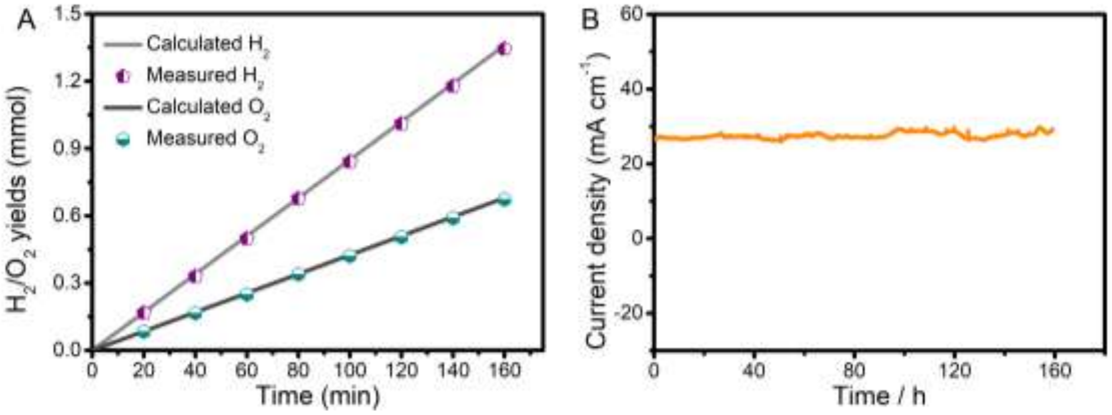

Fig. S22| (A) Experimental H_2 and O_2 production *vs*. theoretical quantities for overall water splitting of CoP_3/Ni_2P-30 in 1 M KOH. (B) The corresponding current-time (*i*-t) curves.

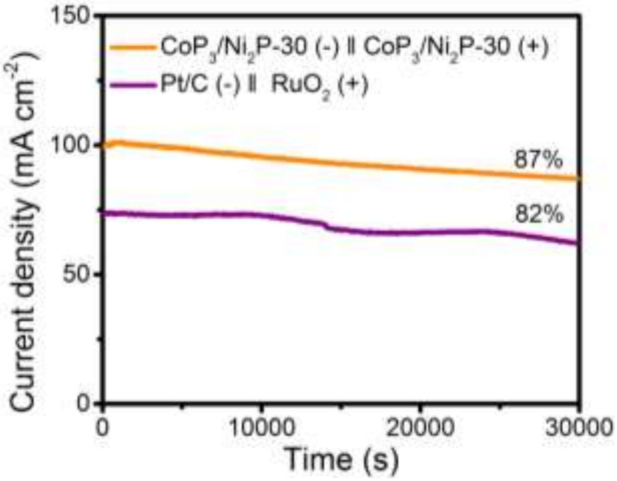

Fig. S23│ The i-t curve for CoP_3/Ni_2P-30 ‖ CoP_3/Ni_2P-30 and Pt/C ‖ RuO_2 in a two-electrode configuration at a potential of 1.80 V.

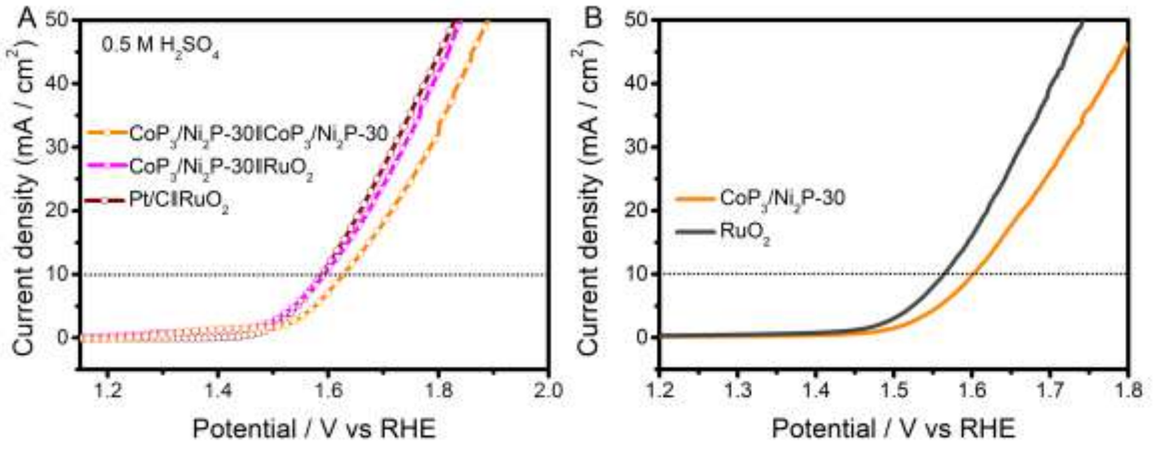

Fig. S24| (A) Polarization curves of CoP_3/Ni_2P-30 ‖ CoP_3/Ni_2P-30, CoP_3/Ni_2P-30 ‖ RuO_2 and Pt/C ‖ RuO_2 coupled catalysts in a two-electrode configuration for overall water splitting in 0.5 M H_2SO_4. (B) Polarization curves of CoP_3/Ni_2P-30 and RuO_2 for OER in 0.5 M H_2SO_4.

As shown in Fig. S24, CoP_3/Ni_2P-30‖RuO_2 exhibits comparable catalytic activity for overall water splitting in acidic media with Pt/C‖RuO_2. But, CoP_3/Ni_2P-30‖CoP_3/Ni_2P-30 exhibits inferior activity to CoP_3/Ni_2P-30‖RuO_2. The poor acidic OER activity of CoP_3/Ni_2P-30 impedes the overall water splitting ability of CoP_3/Ni_2P-30 ‖ CoP_3/Ni_2P-30 in acidic media.

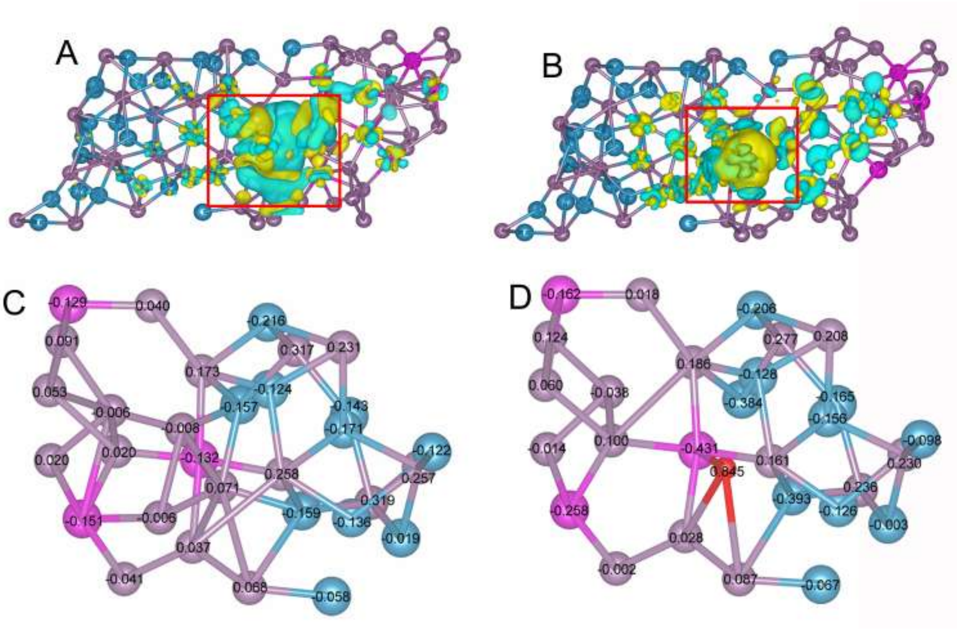

Fig. S25│ (A-B) Calculated charge density differences of CoP_3/Ni_2P (A) and defective CoP_3/Ni_2P (B). The yellow and green regions refer to increased and decreased charge distributions, respectively. (C-D) The bader charge numbers of interfacial atoms in CoP_3/Ni_2P (C) and defective CoP_3/Ni_2P (D).

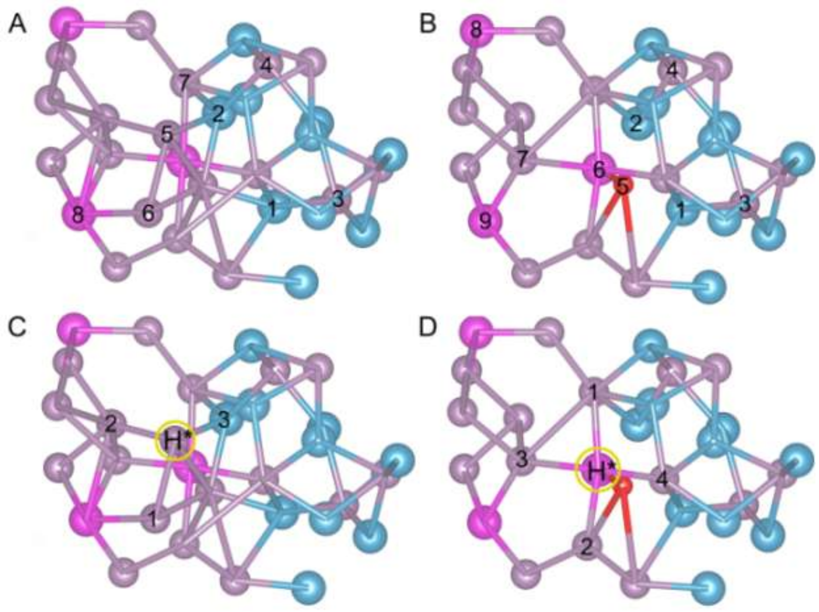

Fig. S26│ The adsorption sites for CoP_3/Ni_2P (A) and defective CoP_3/Ni_2P (B) in acid media, respectively. The adsorption sites of CoP_3/Ni_2P (C) and defective CoP_3/Ni_2P (D) in alkaline media, respectively.

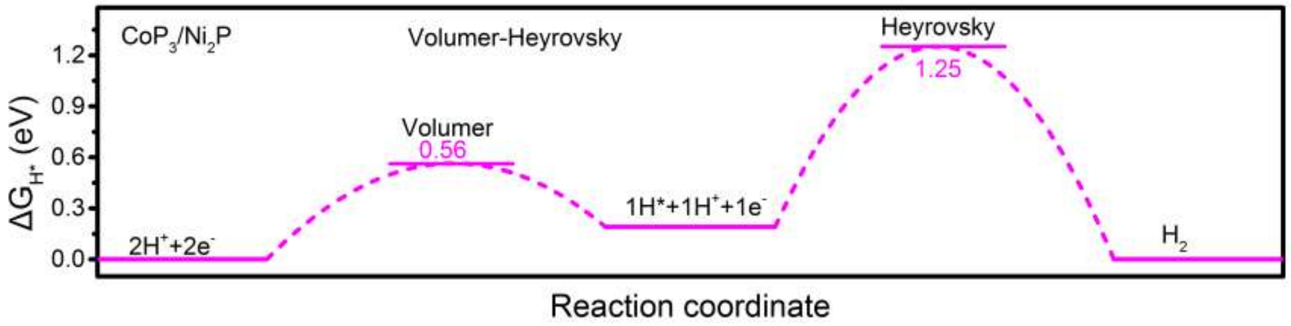

Fig. S27│ The kinetic energy barrier profiles of Volmer-Heyrovsk routes for pristine CoP_3/Ni_2P.

Table S1│The XPS results of CoP_3/Ni_2P and CoP_3/Ni_2P-*t*.

**Sample**	**CoP_3/Ni_2P**	**CoP_3/Ni_2P-15**	**CoP_3/Ni_2P-30**	**CoP_3/Ni_2P-45**
**Co-P/Co-O ratio**	18.40	15.12	13.84	11.85
**Ni-P/Ni-O ratio**	4.50	4.05	3.34	3.01
**Co/Ni-P/P-O ratio**	1.72	1.45	1.30	1.15

Table S2│The EXAFS fitting parameters of Co K-edge for CoP_3/Ni_2P and CoP_3/Ni_2P-30.

**Samples**	**Shell**	**C.N.**	**$\sigma^2(10^{-3}Å^2)$**	**r/Å**
**CoP_3/Ni_2P**	Co-O	3.0	0.7	2.01
	Co-P	6.0	4.9	2.25
	Co-Ni	2. 5	7.9	2.85
	Co-Co	4.0	9.5	3.02
**CoP_3/Ni_2P-30**	Co-O	4.0	1.0	2.03
	Co-P	3.5	1.4	2.27
	Co-Ni	2.5	8.5	2.86
	Co-Co	4.0	10.3	3.07

Table S3│The EXAFS fitting parameters of Ni K-edge for CoP_3/Ni_2P and CoP_3/Ni_2P-30.

Samples	Shell	C.N.	$\sigma^2 (10^{-3} Å^2)$	r/Å
**CoP_3/Ni_2P**	Ni-O	2.0	8.3	2.10
	Ni-P	4.0	10.2	2.25
	Ni-Ni	1.0	2.3	2.61
	Ni-Co	2.0	4.6	2.95
**CoP_3/Ni_2P-30**	Ni-O	5.0	8.0	2.15
	Ni-P	2.5	9.0	2.30
	Ni-Ni	1.0	1.8	2.61
	Ni-Co	2.0	7.5	2.96

Table S4│The electrochemically active surface areas (ECSA) of CoP_3/Ni_2P and CoP_3/Ni_2P-*t*.

sample	CoP_3/Ni_2P	CoP_3/Ni_2P-15	CoP_3/Ni_2P-30	CoP_3/Ni_2P-45	Pt/C
**ECSA (cm^2)**	14.5	10.9	5.4	7.4	19.9

Table S5│The ΔG_{H*} (eV) of different sites for CoP_3/Ni_2P and defective CoP_3/Ni_2P in acid media.

Site	**1**	**2**	**3**	**4**	**5**	**6**	**7**	**8**	**9**
**CoP_3/Ni_2P**	0.38	0.65	0.43	0.23	0.19	0.43	0.98	-0.86	
**CoP_3/Ni_2P-Vp**	0.47	unstable	0.15	0.25	0.54	-0.12	1.03	0.24	0.37
**CoP_3/Ni_2P-Vp-O**	0.51	0.57	0.98	unstable	-0.19	0.07	unstable	0.57	-0.53

Table S6│ The $\Delta G_{H\text{-}OH}$ (eV) for different sites of CoP_3/Ni_2P and defective CoP_3/Ni_2P in alkaline media

Site	**1**	**2**	**3**	**4**
**CoP_3/Ni_2P**	0.96	0.74	0.72	
**CoP_3/Ni_2P-Vp**	0.76	0.86	0.54	
**CoP_3/Ni_2P-Vp-O**	-0.41	0.17	-0.26	0.25

References

[1] G. Kresse, J. Furthmüller, *Phys. Rev. B* **1996**, *54*, 11169.

[2] G. Kresse, J. Furthmüller, *Computational Mater. Sci.* **1996**, *6*, 15.

[3] J. P. Perdew, K. Burke, M. Ernzerhof, *Phys. Rev. Lett.* **1996**, *77*, 3865.

[4] Y. Zheng, Y. Jiao, Y. Zhu, L. H. Li, Y. Han, Y. Chen, M. Jaroniec, S. Z. Qiao, *J. Am. Chem. Soc.* **2016**, *138*, 16174.

[5] J. Zhang, T. Wang, P. Liu, S. Liu, R. Dong, X. Zhuang, M. Chen, X. Feng, *Energy Environ. Sci.* **2016**, *9*, 2789.

[6] T. Wu, M. Pi, X. Wang, D. Zhang, S. Chen, *Phys. Chem. Chem. Phys.* **2017**, *19*, 2104.

[7] P. Cai, J. Huang, J. Chen, Z. Wen, *Angew. Chem. Int. Ed. Engl.* **2017**, *56*, 4858.

[8] A.-L. Wang, J. Lin, H. Xu, Y.-X. Tong, G.-R. Li, *J. Mater. Chem. A* **2016**, *4*, 16992.

[9] Y. Zhao, L. Hu, S. Zhao, L. Wu, *Adv. Funct. Mater.* **2016**, *26*, 4085.

[10] D. Das, K. K. Nanda, *Nano Energy* **2016**, *30*, 303.

[11] N. Jiang, B. You, M. Sheng, Y. Sun, *Angew. Chem. Int. Ed. Engl.* **2015**, *54*, 6251.

[12] W. Liu, L. Cao, W. Cheng, Y. Cao, X. Liu, W. Zhang, X. Mou, L. Jin, X. Zheng, W. Che, Q. Liu, T. Yao, S. Wei, *Angew. Chem. Int. Ed. Engl.* **2017**, *56*, 9312.
